# Divergence of cuticular hydrocarbons in two sympatric grasshopper species and the evolution of fatty acid synthases and elongases across insects

**DOI:** 10.1038/srep33695

**Published:** 2016-09-28

**Authors:** Jonas Finck, Emma L. Berdan, Frieder Mayer, Bernhard Ronacher, Sven Geiselhardt

**Affiliations:** 1 Behavioural Physiology, Department of Biology, Humboldt-Universität zu Berlin, Invalidenstr. 43, 10115 Berlin, Germany; 2 Museum für Naturkunde Berlin, Leibniz Institute for Evolution and Biodiversity Science, Invalidenstr. 43, 10115 Berlin, Germany; 3 Berlin-Brandenburg Institute of Advanced Biodiversity Research (BBIB), Altensteinstr. 6, 14195 Berlin, Germany; 4 Institute of Biology, Freie Universität Berlin, Haderslebener Str. 9, 12163 Berlin, Germany

## Abstract

Cuticular hydrocarbons (CHCs) play a major role in the evolution of reproductive isolation between insect species. The CHC profiles of two closely related sympatric grasshopper species, *Chorthippus biguttulus* and *C. mollis*, differ mainly in the position of the first methyl group in major methyl-branched CHCs. The position of methyl branches is determined either by a fatty acid synthase (FAS) or by elongases. Both protein families showed an expansion in insects. Interestingly, the FAS family showed several lineage-specific expansions, especially in insect orders with highly diverse methyl-branched CHC profiles. We found five putative FASs and 12 putative elongases in the reference transcriptomes for both species. A dN/dS test showed no evidence for positive selection acting on FASs and elongases in these grasshoppers. However, one candidate FAS showed species-specific transcriptional differences and may contribute to the shift of the methyl-branch position between the species. In addition, transcript levels of four elongases were expressed differentially between the sexes. Our study indicates that complex methyl-branched CHC profiles are linked to an expansion of FASs genes, but that species differences can also mediated at the transcriptional level.

Cuticular hydrocarbons (CHCs) are omnipresent on the surface of insects and play a dual role in waterproofing and in chemical communication^1^. In many insect species, CHCs are regarded as a central component of mate recognition systems and thus contribute to behavioral isolation between species^2^,^3^,^4^,^5^. Insects have evolved a vast number of CHCs (>1000) differing in chain lengths, number and position of double bonds and methyl groups, respectively^6^,^7^. Comparative studies have demonstrated that CHC profiles tend to be species-specific mixtures ranging in complexity from a couple to more than a hundred compounds^8^,^9^.

The fundamentals of the CHC biosynthesis in insects are well established^10^. The majority of CHCs are synthesized *de novo* in oenocytes by a sophisticated network of fatty acid synthases (FASs), elongases, desaturases, reductases, and a decarbonylase^10^,^11^,^12^. Methyl-branched CHCs result from the incorporating of methylmalonyl-CoA instead of malonyl-CoA early during chain elongation by a microsomal FAS^10^,^12^. Animal FASs are single multifunctional enzymes consisting of two identical monomers^13^,^14^. The FAS monomer contains seven distinct functional domains in the following order (from the N-terminus): β-ketoacyl synthase (KS), malonyl-/acetyl transferase (MAT), β-hydroxyacyl dehydratase (DH), enoyl reductase (ER), β-ketoacyl reductase (KR), acyl carrier protein (ACP), and thioesterase (TE). In most biological systems, the major product released by FASs is palmitic acid (C16:0)^10^,^13^,^14^. Subsequently, palmitic acid is further elongated to very long-chain fatty acids by members of the elongase family, characterized by the ELO domain (PF01151; GNS1/SUR4 family), with a conserved LHXXHH histidine box motif^15^. Despite our basic knowledge about the biosynthesis and composition of many CHC profiles (phenotypes) in a broad range of insect taxa we lack understanding of how new phenotypes may evolve.

The evolution of novel phenotypes can have different molecular origins^16^. Modified gene expression patterns caused by alterations in either *cis*-regulatory sequences or *trans*-acting transcription factors can give rise to novel phenotypes^17^,^18^. In addition, coding sequence changes of preexisting genes and/or gene duplications can also lead to modifications of existing phenotypes. Gene duplications are generally considered as a major source of evolutionary innovations^19^,^20^,^21^,^22^,^23^. Duplication of a gene causes functional redundancy that hampers a stable maintenance of two functionally redundant genes in the genome^24^. Consequently, the two paralogs have different evolutionary fates^19^. Most likely, functional redundancy results in pseudogenerization, as one paralog is freed from purifying selection and can accumulate deleterious mutations over time^19^,^20^. Nevertheless, in some cases, the accumulation of neutral mutations may lead to the origin of novel functions, i.e. neofunctionalization, and the evolution of novel phenotypes. The origin of species-specific CHC profiles in insects can be the result of mutation in genes which are involved in the biosynthetic pathway of CHCs^25^. However, the relative importance of regulatory changes and gene duplications for the origin of novel CHC phenotypes has rarely been investigated.

Here, we use two closely related and morphologically cryptic grasshopper species, *Chorthippus biguttulus* and *C. mollis*, (Orthoptera, Acrididae, Gomphocerinae) to elucidate molecular mechanisms underlying the divergence of CHC profiles in closely related insect species. These grasshoppers have traditionally been used as model organisms for studying acoustic communication as they produce species-specific calling songs that are reliable signals for species identification^26^,^27^,^28^,^29^,^30^. However, several studies suggest that chemical communication via CHCs can also play a crucial role in mate recognition in the genus *Chorthippus*^31^,^32^. Thus, chemical and acoustic communication might be equally important in species and mate recognition in grasshoppers, as already shown for crickets (Orthoptera; Gryllidae)^33^,^34^,^35^,^36^,^37^.

In this study, we first determined whether CHC profiles (phenotypes) have diverged between the sexes and species in two Gomphocerinae species, *Chorthippus biguttulus* and *C. mollis*. Second, we identified candidate genes for FASs and elongases in *Chorthippus* transcriptomes as these genes are involved in regulation of CHC chain length and the position of methyl-branches. Third, we used these candidate genes to examine (i) differential expression patterns between the sexes as well as between *C. biguttulus* and *C. mollis*, (ii) coding sequence changes, and (iii) sequence evolution on a broad scale (class Insecta).

## Results

### Composition of cuticular hydrocarbons

The CHC profiles were mixtures of *n*-alkanes and mono-, di- and trimethyl-branched alkanes (Me-, diMe-, triMeCHCs) with carbon backbones ranging from C_25_ to C_39_. *n*-Alkanes and methyl-branched alkanes were equally abundant (Supplementary Table S1). The *n*-alkane fraction consisted of a homologous series ranging from C_25_ to C_33_, with *n*-nonacosane (*n*-C29) as the dominant compound in both species. In contrast to the uniform composition of *n*-alkanes, both species differed considerably in the composition of their methyl-branched alkanes. In general, the position of the first methyl-branch is shifted by 2 carbon units between the species, i.e. from positions 11 and 13 in *C. biguttulus* to positions 13 and 15 in *C. mollis* (Supplementary Table S2). Nevertheless, some *C. biguttulus* individuals showed the branching pattern typical for *C. mollis*.

### Species and sex differences in CHC composition

The final dataset for the comparison of the cuticular hydrocarbon (CHC) phenotypes of *C. biguttulus* and *C. mollis* comprised 34 different peaks (those that were present in at least 10 individuals; Table S1). The number of peaks per individual was consistent across species and sexes (*C. biguttulus* females: 16.7 ± 1.8 (N = 40); males: 16.9 ± 1.6 (N = 34); *C. mollis* females: 16.1 ± 1.9 (N = 17); males: 16.9 ± 1.1 (N = 34)).

To assess quantitative differences of the hydrocarbon profiles we performed a principal component analysis (PCA) using the relative composition of the CHC profiles. The first five principal components together explained 71.3% of total variance in the CHC phenotypes (PC1 = 39.7%, PC2 = 14.5%, PC3 = 8.6%, PC4 = 4.7%, PC5 = 3.9%).

**Figure 1:**
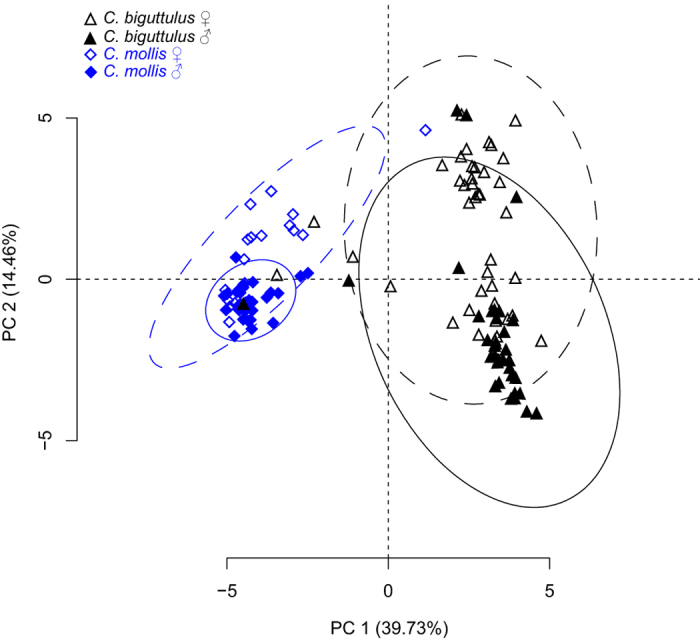
Principal component analysis (PCA) of cuticular hydrocarbon (CHC) phenotypes of male and female *Chorthippus biguttulus* and *C.* mollis. Shown are principal component (PC) 1 *versus* 2 with variances explained by each PC given in parentheses. Ellipses indicate 95% confidence intervals. The PCA is based on the relative composition of 34 CHC peaks (see Table 2 for loadings).

PC1 (39.7%) clearly separated the species, while PC2 (14.5%) separated individuals according to sex (Fig. 1, Table 1). A multivariate analysis revealed a significant effects of species, sex and the interaction between the two (species: *F*(5, 117) = 145.977, p < 0.001; sex *F*(5, 117) = 13.842, p < 0.001; interaction of species and sex: *F*(5, 117) = 11.936, p < 0.001). Linear models showed that PC1, PC3 and PC4 differed significantly between species, while males and females differed significantly in PC2 and PC3 scores (Table 1). We also observed a significant species × sex interaction in all principal components (Table 1). The PC2 interaction is due to a stronger separation between the sexes in *C. biguttulus* and the PC1, PC3, and PC4 interaction is due to the fact that males and females of *C. mollis* were more strongly separated in comparison to *C. biguttulus* (Fig. 1, Supplementary Fig. S1). The compounds that contributed most to PC1 were diMeCHCs (Table 2), with negative factor loadings for 15,x-diMeCHCs (indicative for *C. mollis*) and positive factor loadings for 13,x-diMeCHCs (indicative for *C. biguttulus*). The CHC profiles between the sexes differed mainly in the relative amount of triMeCHCs and diMeC35 (peaks 18 and 19). Females exhibited a greater proportion of 11,x-/9,x-/7,x-diMeC35 (peak 19) and 11,x,x-/9,x,x-triMeCHCs (peaks 22 and 31), while males have higher proportions of 13,x-/11,x-/9,x-diMeC35 (peak 18) and 13,x,x-/11,x,x-triMeCHCs (peaks 21 and 30). Similar to the differences between species, the sexes differed mainly in the position of the first methyl-branch of the major CHCs, i.e. shifted by two carbon units between the species (Supplementary Table S2).

### Ortholog assignment of fatty acid synthases and elongases in *Chorthippus*

**Figure 2:**
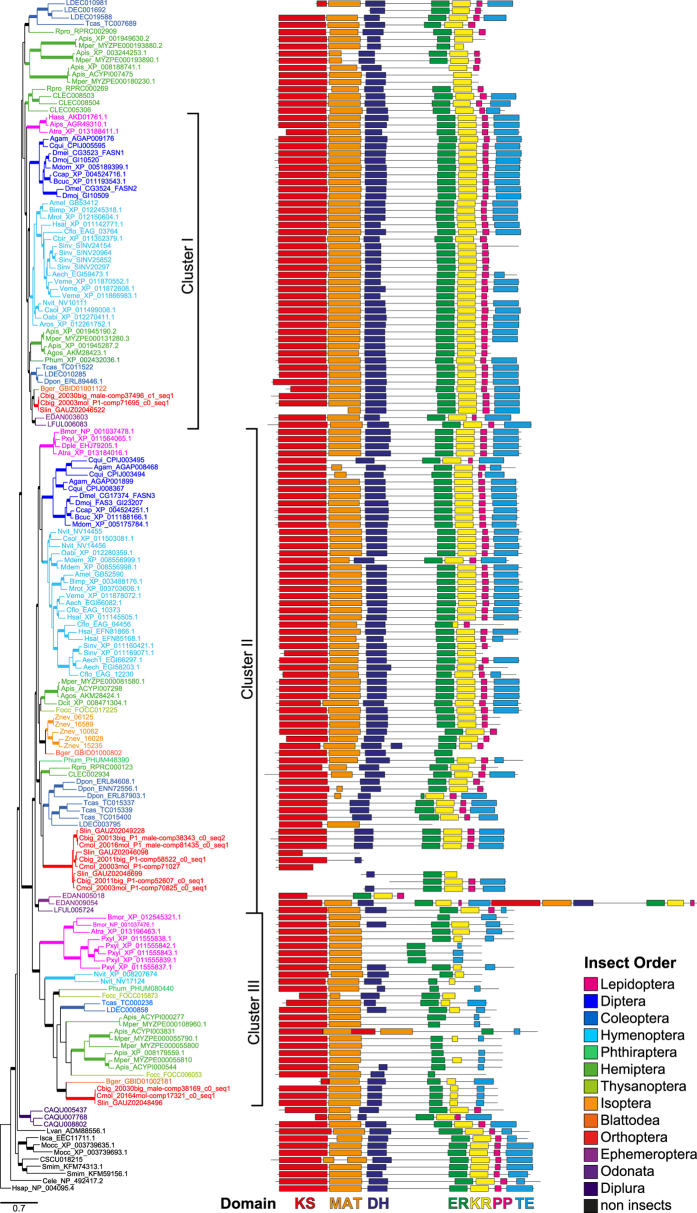
Phylogenetic relationship and domain structure of fatty acid synthases in insects. The maximum-likelihood tree was computed based on 164 protein sequences from 45 insect species of 13 insect orders and seven non-insects outgroup species from three different phyla. The tree is rooted with the human FAS. Bold branches indicate a LRT support values ≥0.9. The four letters code indicate the species, followed by the Genbank accession numbers (see Supplementary Table S5 for details).

We identified five transcripts coding for putative FASs in both *Chorthippus* reference transcriptomes. The assignment of orthologous genes between both *Chorthippus* species resulted in five ortholog pairs (Table 3). The similarities of coding nucleotide and protein sequences, respectively, within ortholog pairs were >98.6% and 99.2%. One ortholog pair (cluster I, Table 3) was assigned as ortholog to FASN1 (CG3523) in *D. melanogaster*, while all other FAS ortholog pairs in *Chorthippus* had no reciprocal best hit with a FAS in *D. melanogaster*. However, each ortholog pair in *Chorthippus* has a corresponding ortholog in the gomphocerine grasshopper *Stenobothrus lineatus* (Fig. 2).

The domain structure analysis revealed that only one ortholog pair (cluster I) contained the full open reading frame (ORF) with all seven functional domains. The other ortholog pairs lacked certain domains, showed truncated domains or contained incomplete ORFs (Fig. 2). Two related ortholog pairs (cluster II-a/c) lacked the MAT domain and another closely related ortholog pair (cluster II-b) had an incomplete ORF that contained only the C-terminal domains. In *C. mollis*, two FAS transcripts with incomplete ORFs (cluster II-b/c) had short overlapping ends (11 AA) with identical protein sequences, which might be a hint that both transcripts belong to a single gene (Fig. 2). All FAS sequences in cluster III lacked the PP domain and showed modification in DH, ER, KR, and TE domains, but not in the KS and MAT domain (cluster III, Fig. 2, Table 3). A phylogenetic analysis based on the KS or MAT domain revealed higher sequence similarity of MAT and KS domains of cluster II and III within a lineage than between sequences of cluster III from divergent insect orders (Fig. 2, Supplementary Fig. S2).

**Figure 3:**
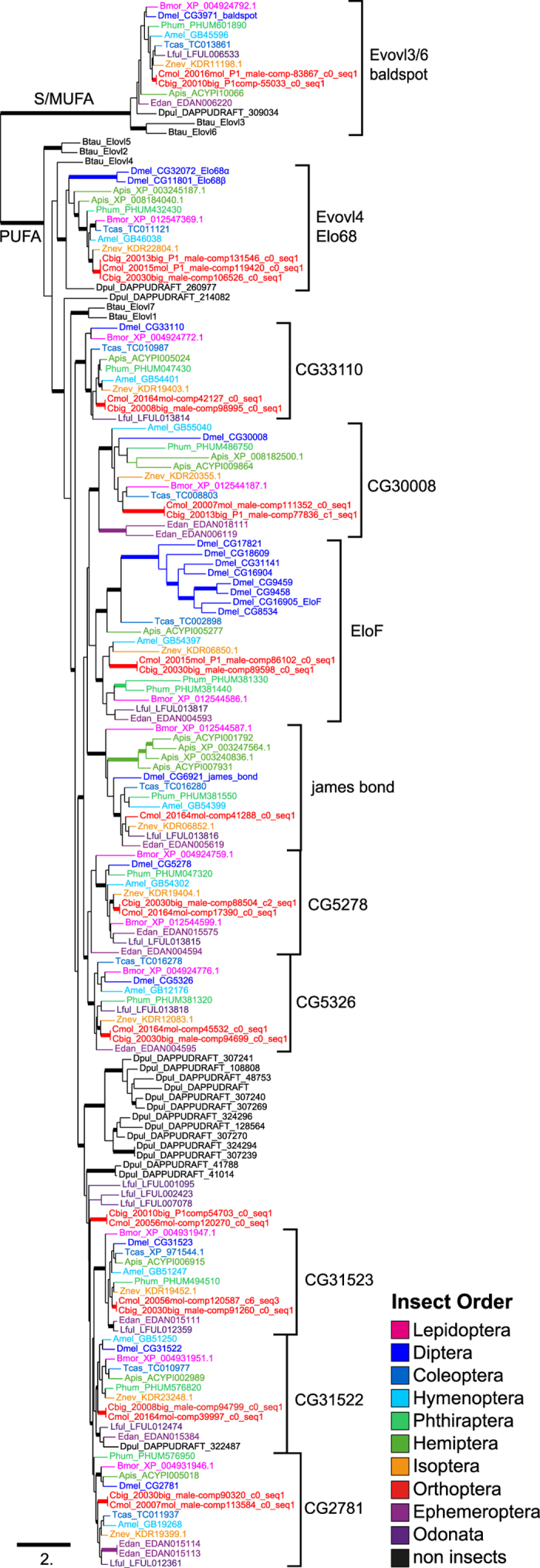
Phylogenetic relationship of the fatty acid elongase family in insects. The unrooted maximum-likelihood tree was computed based on 159 protein sequences from representatives of ten different insect orders, a crustacean, and a mammalian outgroup species. Bold branches indicate a LRT support values ≥0.9. Clusters are named after the *Drosophila melanogaster* ortholog. The four letter codes indicate the species; followed by gene names or accession numbers (see Supplementary Table S5 for details).

Using the elongase genes from *D. melanogaster*, a tblastn search resulted in 12 transcripts coding for putative elongases in each *Chorthippus* reference transcriptome. Both *Chorthippus* species shared 11 ortholog pairs, only two transcripts had no corresponding ortholog in the other species (Table 3). In the first case, *C. biguttulus* had two paralogs in the Elo68 cluster while *C. mollis* had only a single copy (Fig. 3). However, the coding sequences of all three transcripts were identical; the 3′ non-coding region of the mRNA differed between the two paralogs in *C. biguttulus* and allowed an ortholog assignment of the *C. mollis* transcript. In the second case, *C. biguttulus* lacked the ortholog to CG6921 (james bond). All putative elongase transcripts of *Chorthippus* species could be assigned to orthologs in *D. melanogaster*, except for a single ortholog pair (Fig. 3, Table 3).

### Signature of selection analysis

We calculated dN/dS ratios for nine ortholog pairs (Supplementary Table S3). Four ortholog pairs showed either no nonsynonymous or no synonymous substitutions, and three sequences had no SNPs. The signature of selection analysis provided no evidence for positive selection acting on FAS and elongases in the two *Chorthippus* species. The dN/dS ratios of ortholog pairs ranged from 0 to 0.129, indicating that purifying selection acts on these genes (Supplementary Table S3).

### Evolution of fatty acid synthases (FASs) and elongases in insects

The majority of FASs of pterygote insects showed two distinct clusters with regular FASs that contain all seven functional domains (clusters I and II; Fig. 2), while the three FAS copies of the two-pronged bristletail *Catajapyx aquilonaris* (Diplura) formed a single cluster that represents the sister clade to all other insect FASs (Fig. 2).

In most cases, each of the two clusters contained a single copy of a regular FAS per species. However, the termite *Zootermopsis nevadensis* (Isoptera) and ants showed a noticeable increase in copy numbers in cluster I and II, e.g. the red imported fire ant (*Solenopsis invicta*) (Hymenoptera) possesses six FAS genes. However, other insect orders, like Orthoptera, Hemiptera, Coleoptera, and Lepidoptera, showed expansions of ‘aberrant’ FASs that either lack certain domains or show an unusual domain structure. The five FAS ortholog pairs of *Chorthippus* were allocated to three different clusters (Fig. 2). The ortholog pair in cluster I contained all seven functional domains, while the three ortholog pairs in cluster II either lacked the MAT domain or had incomplete open reading frames. The last ortholog pair fell in cluster III and was characterized by an unusual domain structure.

The basal split of the elongase phylogeny separated the two previously characterized S/MUFA and PUFA elongase subfamilies^15^ (Fig. 3). The basal S/MUFA cluster (*baldspot*) contained the insect orthologs of the vertebrate ELOVL3 and ELOVL6 genes, with 43% and 51% protein sequence similarity between *Chorthippus* and cattle (*Bos taurus*). All other elongases belonged to the PUFA subfamily. We found ten distinct ortholog clusters in the PUFA subfamily; most of them contained a single elongase copy for each of the studied insect orders. However, *D. melanogaster* and the pea aphid *Acyrthosiphon pisum* showed a large increase in copy number in the EloF (8 copies) and james bond (4 copies) cluster, respectively. Eight of the 10 PUFA elongase clusters appeared to be insect-specific. Only the *Elo68* and CG31522 cluster contained orthologs from non-insect outgroup species. With exception of the ortholog pair in the CG30008 cluster, each ortholog pair of *Chorthippus* clustered exactly in the ortholog cluster predicted by the ortholog assignment.

### Differential expression of candidate fatty acid synthases and elongase genes

Among the 5 FAS and 11 elongase ortholog pairs of *Chorthippus* species, we found only a single FAS ortholog pair (cluster II-b) that was differentially expressed between both species, with a 2.9-fold higher expression in *C. biguttulus* (Table 4). However, the expression levels of this FAS transcript differed not only between species, but also strongly between the sexes (7.6-fold higher in females). In addition, we found two other FASs and three elongases that had significantly higher expression in males than in females (Table 4). The two putative FAS transcripts (cluster II-a and III) showed higher expression in males (8.4 fold higher in *C. biguttulus* and 2.4-fold higher in *C. mollis*). The strong differences between the male-biased expression of the FAS cluster II-a transcripts, resulted in a significant species × sex interaction term. Of the three differentially expressed elongases, the CG30008 orthologs showed the strongest male-biased expression (23.2-fold). The other two elongases had 2.3-fold (CG16905) and 3.6-fold (CG5326) higher expression in males.

## Discussion

In addition to their divergent acoustic signals, the sympatric *Chorthippus* grasshopper species, *C. biguttulus* and *C. mollis*, differed significantly in their CHC profiles. The CHC profiles of both species consisted of a series of *n*-alkanes, followed by a series of various methyl-branched alkanes. Our study demonstrated that *C. biguttulus* and *C. mollis* as well as males and females of both species show quantitative differences in their CHC phenotypes. Both the general pattern of hydrocarbons with series of *n*-alkanes and methyl-branched alkanes and the interspecific variation based on quantitative rather than qualitative differences seemed to be relatively conserved throughout the family Acrididae^38^,^39^,^40^,^41^,^42^. The most striking difference between the two species was the shift of the first methyl-branch position in multimethyl-branched CHCs, i.e. position 13 in *C. biguttulus* and position 15 in *C. mollis*. However, *C. biguttulus* also showed large variability in CHC phenotype, with some individuals exhibiting the methyl-branching pattern typical for *C. mollis*. These individuals clustered together with *C. mollis* in the PCA, illustrating that without this shift, both species are nearly indistinguishable based on their CHC phenotypes. Methyl-branches are incorporated during the fatty acid elongation process by FASs and/or elongases^10^. Thus, we focused on these protein families as candidates for producing the species and sex specific CHC pattern.

We found numerous candidate FAS transcripts in *Chorthippus* that cluster into three groups on our FAS phylogeny. In general, the phylogenetic analysis of the FAS family indicates a duplication of the ancestral FAS gene in the common ancestor of pterygote insects. Thus, most insect orders possess at least two FAS gene copies. This ancestral duplication resulted in two clusters with regular FASs that contain all seven functional domains (cluster I and II). In many insects orders, these two copies underwent additional lineage-specific duplication events resulting in independent expansions of the FAS family (paralogs: Orthoptera: ≥4 (*Chorthippus*); Isoptera: 5 (*Z. nevadensis*); Heteroptera: 11 (*A. pisum*); Hymenoptera: 6 (*S. invicta*); Coleoptera: 6 (*T. castaneum*); Lepidoptera: 6 (*P. xylostella*)). Although the two-pronged bristletail *C. aquilonaris* (Diplura) has also multiple FAS copies, the three paralogs formed a single cluster that is the sister clade to the FASs of pterygote insects.

Some FAS copies showed significant deviations from the classical FAS domain structure. A frequent modification that evolved independently in at least three insect orders (Orthoptera, Coleoptera, Diptera) was the loss of the MAT domain in cluster II FASs. Two such FAS transcripts (cluster II-a and II-b) in *Chorthippus* showed sex-biased expression but in opposite directions, i.e. male-biased in FAS II-a and female-biased in FAS II-b. In addition, the FAS transcript FAS II-b showed indications for differential expression between the species and might be a potential candidate involved in the generation of the divergent CHC profiles of these grasshopper species. The FAS II-a was previously identified in a population genomic scan on *C. biguttulus* and *C. mollis*, indicating that this locus is under selection^43^. Looking at the coding sequence we found one non-synonymous substitution, but no significant evidence for positive selection (dN/dS = 0.103). All the *Chorthippus* sequences of cluster II lack the MAT domain. This domain is responsible for substrate recruitment and loading^14^. Thus, it is unclear whether these transcripts code for functional proteins. However, in *Tribolium castaneum*, an RNAi knockdown of TC15337, that also lacks the MAT domain, leads to a mortality of 60% and 40% after larval and pupal injection, respectively^44^. This suggests that TC15337 codes for a functional protein, but it is yet unknown whether it codes for a FAS or another protein.

The grasshopper FAS transcript of the third cluster III showed female-biased expression. This FAS exhibit modifications in the DH, ER, KR, PP, and TE domains that were either truncated or completely lost. A putative FAS in *T. castaneum* (TC000238) has a very similar domain structure and RNAi knockdown implies that this protein is functionally active (100% mortality after larval injection)^44^. All FAS sequences in cluster III had modifications in one or more domains, with the exception of the KS and MAT domain. In order to examine whether these FASs are derived from a common ancestor sequence or were derived by lineage-specific duplication of FASs, we looked at the phylogeny of the KS and MAT domains separately (Supplementary Fig. S2). The results of the phylogeny of these domains, together with the inconsistencies of domain modification of the FASs in cluster III, indicates that these genes are most likely derived independently by lineage-specific duplications from FASs in cluster II.

Our FAS phylogeny has shown that many insect groups showed lineage-specific expansions of FAS copies, especially ants, beetles, and aphids. Ants and beetles are known for their highly diverse and complex CHC profiles. Almost 1000 different CHCs have been characterized in ants, of which dimethyl-branched alkanes is the largest group (>600 compounds)^6^, and beetles show a similar diversity of methyl-branched CHCs^7^. Thus, the expansions of FAS copies observed in some insect groups might be an indication that multiple FASs are involved in the generation of such a great CHC richness.

Early studies on the fatty acid biosynthesis in insects^45^,^46^,^47^ and vertebrates^48^,^49^ suggest that a single FAS can synthesize both straight-chain and methyl-branched fatty acids. FASs of the bug *Triatoma infestans* (Hemiptera)^46^, the housefly *Musca domestica*^47^, and the fruit fly *D. melanogaster*^45^ can incorporate both malonyl-CoA and methylmalonyl-CoA during chain elongation, resulting in methyl-branched fatty acids. However, a recent study of CHC biosynthesis in *Drosophila* indicates that methyl-branched CHCs are synthesized by a special FAS^12^. The genome of the fruit fly *D. melanogaster* contains three FAS paralogs: FASN1 (CG3523), FASN2 (CG3524), and FASN3 (CG17374). FASN1 is expressed in the fat body, while FASN2 and FASN3 are both expressed in oenocytes of adult flies^12^. The microsomal FASN2 uses isobutyryl-CoA as primer, instead of acetyl-CoA, and is responsible for the production of 2-MeCHCs, the major components of the CHC profile in *D. melanogaster*. Our FAS phylogeny demonstrated that FASN2 originated from a *Drosophila*-specific gene duplication in cluster I, indicating that the biosynthesis of methyl-branched CHCs in *Drosophila* is a result of gene duplication and neofunctionalization. Consequently, FASs involved in the biosynthesis of 2-MeCHCs or other methyl-branched CHCs in other insects must have evolved independently. This multiple origin of methyl-branched CHC biosynthesis might explain the multiple independent expansions of FASs in different insect orders observed in our FAS phylogeny.

The regular FASs release fatty acids with chain length up to 16, with palmitic acid (C16:0) as major product^10^. Thus, the production of long-chained CHCs depends on elongases that elongate the medium-chain fatty acids to very-long chain fatty acids. The elongase family comprises two subfamilies, the S/MUFA and the PUFA subfamily^15^. Members of the S/MUFA subfamily are thought to elongate saturated and monounsaturated fatty acids, while members of the PUFA subfamily elongate polyunsaturated fatty acids. However, this classification is largely based on functional characterization in mammals, whereas the specificity of elongases in insects needs not fit into this classification^50^.

In insects, the S/MUFA subfamily forms a single highly conserved cluster (*baldspot*) and the PUFA subfamily is expanded into ten distinct clusters, of which eight were insect specific (Fig. 3). In contrast to FASs that showed multiple lineage-specific duplications, elongase clusters contained only a single copy per insect species, except for the EloF and james bond cluster that showed expansions in *D. melanogaster* and *A. pisum*, respectively. Beside the 11 elongases in these ancient clusters, *Chorthippus* grasshoppers possess an additional copy without an ortholog in other insect orders. The expression pattern of elongases was similar in both *Chorthippus* species, but three elongases (EloF, CG30008, and CG5326 orthologs) showed male-biased gene expression. Interestingly, in *D. melanogaster* the EloF (CG16905) gene shows female-biased expression and is involved in the biosynthesis of sexually dimorphic CHC profiles^51^. Fruit fly males have CHCs with chain length of C23 and C25 and females with C27 and C29. RNAi knockdown of EloF induced a decrease of C29dienes and an increase of C25dienes. In contrast, the CG18609 gene (EloF cluster) shows a male-biased expression^52^ and a RNAi knockdown results in a strong decrease of total CHCs in males but not in females^53^. In the honeybee, *Apis mellifera*, two elongases, GB54399 and GB40681, are positively correlated with the production of methyl-branched CHCs^50^. The expression of GB54399 (*james bond* ortholog) is correlated with monomethyl-branched CHCs, while GB40681 (CG30008 ortholog) is highly correlated with dimethyl-branched CHCs (11,15-diMeC27, 9,13-diMeC29, 3,7-diMeC31). Thus, the male-biased expression in the EloF and CG30008 orthologs makes both genes candidates for the biosynthesis of a higher proportion of diMeC35 in males of *C. biguttulus* (3.0-fold) and *C. mollis* (1.7-fold).

In conclusion, we demonstrated that the CHC profiles of the grasshopper species, *C. biguttulus* and *C. mollis,* differ in the first methyl-branch position in multimethyl-branched CHCs. The high sequence similarity of ortholog pairs and the absence of positive selection acting on FAS and elongase genes in *Chorthippus* species suggest that the variation in CHC profiles in closely related species is mainly mediated at the transcriptional level. Similar conclusions can be drawn from the *Drosophila* sister species *D. serrata* and *D. birchii*^12^. Both species have a functional FASN2 gene, responsible for the biosynthesis of 2-MeCHCs, but *D. birchii* has lost the FASN2 expression in oenocytes, due to cis-regulatory changes. However, the research about the biosynthesis of internally methyl-branched CHCs and its transcriptional regulation is still in its infancy. Although several hundreds of methyl-branched CHCs are known from insects, the enzymatic machinery behind this diversity is largely unknown. In particular, we need a better functional characterization of the FAS and elongase families in insects. Our study has shown that both the FAS and elongase family exhibit an increase in copy numbers in insects. However, the evolutionary histories of both protein families are distinct different. The elongase family has undergone a rapid expansion in the ancestor of insects resulting in eleven paralogs of which eight are insect specific. After this ancestral expansion, the copy numbers did not further increase, with some exceptions. In contrast, the FAS family showed only a single duplication in the ancestor of pterygote insects that was followed by multiple independent lineage-specific expansions. Interestingly, insect groups known for a high diversity of methyl-branched CHCs, as ants or beetles, have high numbers of FAS copies. At least in *Drosophila*, the biosynthesis of methyl-branched CHCs can be linked to duplication and neofunctionalization of a FAS gene. However, it remains to be tested whether diversification of methyl-branched CHCs is really driven by the expansion of FAS genes.

## Methods

### Insects and rearing conditions

For the chemical analyses, *C. biguttulus* was collected at Wendebachstausee near Göttingen, Lower Saxony (N51°28′10.41, E9°56′24.98), and *C. mollis* was collected in Alterlangen, Bavaria (N49°36′35.18, E10°59′3.05) in July and August 2013. For genetic analysis, we used 12 individuals of each species originating from two populations (three males and three females per population), Alterlangen collected in August 2013 and Neuenhagen near Berlin (N52°32′3.33, E13°40′23.01) collected in September 2012 and 2013 (Table S4).

All individuals were caught as late instar nymphs (3^rd^ & 4^th^) and were subsequently kept in a common room at 25–30 °C, 25–30% relative humidity, and a 16:8 h light:dark cycle. Grasshoppers were fed *ad libitum* with a mixture of different grasses (*Festuca rubra rubra, Dactylis glomerata, Poa pratensis*) (seeds from Revierberatung Wolmersdorf Nindorf, Germany). After the final molt, individuals were separated by sex to ensure virginity.

Individuals used for RNA extraction were killed by decapitation within 7 days after their final molt, their gut was removed, and they were stored in liquid nitrogen or in RNAlater (Qiagen, Limburg, Netherlands), due to storage capacity in the liquid nitrogen tank. For RNAlater storage, samples were cut into pieces and incubated in RNAlater at 4 °C overnight, the tissue was removed from the RNAlater and stored at −80 °C. Although collection dates, populations, and preservation methods vary, both species were always caught together. Therefore species differences are not affected by difference in collection date, population or preservation method.

### Extraction of cuticular hydrocarbons

Grasshoppers were frozen at −20 °C four to six days after their final molt. Grasshoppers were thawed for 15 min at room temperature before hydrocarbons were extracted by immersing an individual in 1 ml of *n*-hexane for 5 min^41^. Samples were stored at −20 °C until further analysis. Cuticular extracts were concentrated under a gentle stream of nitrogen to a volume of 100 μl. A blank hexane sample was treated the same way to control for potential contamination of samples.

### Chemical analysis

In order to examine species or sex specific difference in CHC profile, chemical identification of cuticular extracts was performed on a coupled gas chromatograph-mass spectrometer (GC-MS) system (7890A GC–5975C MSD; Agilent, Waldbronn, Germany) equipped with an Agilent 7693A automatic liquid sampler for injection. An aliquot of 1 μl of each sample was injected in splitless mode at 300 °C. A fused silica column (ZB-5HT Inferno, 30 m × 0.25 mm × 0.25 μm, Phenomenex Inc., Torrance, CA, USA) was used for separation with a constant helium flow of 1 ml/min. The oven temperature program was started at 100 °C and then heated to 320 °C at a rate of 10 °C/min (20 min isotherm). Electron impact ionization was 70 eV.

Hydrocarbons were identified by their mass spectra and corroborated by their retention indices^54^. Peak areas relative to total peak area were computed for each compound, and peaks that occurred in less than 10 individual CHC profiles were discarded from further analyses. Prior to multivariate statistics, the data were transformed as follows: *z*_*ip*_ = ln[*A*_*ip*_*/g*(*A*_*p*_)], where *A*_*ip*_ is the area of peak i for individual p, *g(A*_*p*_) is the geometric mean of all peaks for individual p, and *z*_*ip*_ is the transformed area of peak i for individual p^55^. As the logarithm is not defined for zero values, a constant of 0.01 was added to each relative peak area^56^.

As internally branched alkanes (first methyl-branch at position ≥9) could not be sufficiently separated by GC, we used the ratios of the peak heights of their diagnostic fragment ions, i.e. m/z 140 (position 9), m/z 168 (position 11), m/z 196 (position 13), and m/z 224 (position 15), as an approximation to the relative composition of the respective methyl-branched alkanes (Supplementary Table S2).

### Statistical analysis

For quantitative comparisons of the CHC phenotypes, a Principal Component Analysis (PCA) was performed on 34 variables (peaks) and 125 individuals using “FactoMineR” package^57^ in R^58^. By using the PC scores for each individual on PC 1–5 we tested for differences between the two species, the sexes within species and the interaction of species and sex. We first ran multivariate analysis of all five PCs and then continued the statistical analysis by running 5 linear models with the pc scores as dependent variable and species and sex as explanatory variables with the interaction of species × sex in R with the lm() function. Tukey’s HSD *post hoc* tests were used for pairwise comparisons of males and females within a species and across species with TukeyHSD() function. All analyses were performed in R (version 3.2.2).

### Identification and ortholog assignment of fatty acid synthases and elongases in *Chorthippus*

We took a transcriptomic approach to identify candidate genes for CHC synthesis. Based on a literature search, 22 reference protein sequences from *Drosophila melanogaster* related to CHC biosynthesis (i.e. 3 FASs and 19 elongases) were downloaded from FlyBase (http://flybase.org) (Supplementary Table S5). In order to identify homologs in *Chorthippus* grasshoppers, we used tblastn to compare our set of 22 reference proteins to a reference transcriptome of *C. biguttulus* and *C. mollis* respectively (Mayer *et al*. unpublished). We retained up to 10 hits per protein with a cut-off e-value of 10^−5^. Best hit transcripts (putative homolog) for each candidate were determined based on highest sequence identity and lowest e-value. Orthologs were then assigned by reciprocal best hits, using the *C. biguttulus* and *C. mollis* candidates^59^.

### RNA extraction and sequencing

We wanted to determine if any of our candidate genes were differentially expressed between sexes or species. We collected 12 individuals of each species originating from two populations (three males and three females per population). Whole body samples were individually homogenized in TriFast using a MINILYS homogenizer with the Precellys ceramic kit (1.4/2.8 mm) (all from peqlab, VWR International GmbH, Erlangen, Germany). Total RNA was extracted from the samples following the manufacturer’s instructions (for peqGOLD TriFast) except that samples that had been stored in RNAlater were precipitated with isopropanol that had been diluted 1:2 with nuclease free water. All total RNA samples were checked for purity and quality using a NanoDrop spectrophotometer (NanoDrop Products, Wilmington, DE, USA) and a 2100 Bioanalyzer (Agilent Technologies, Santa Clara, CA, USA). Total RNA samples were determine as pure with a 260/280 value of ~2.0 and a slightly higher 260/230 value associated. If total RNA samples showed strong differences in absorbance, a re-extraction with 1 ml peqGOLD TriFast was performed. All samples showed no visible RNA degradation at Agilent RNA 6000 Pico Assay electropherogram. For mRNA isolation and to decrease ribosomal RNA contamination, an mRNA enrichment was performed using the Dynabeads mRNA Purification Kit (Life Technologies, Carlsbad, CA, USA).

For Illumina sequencing, we prepared directional, strand specific RNA libraries using the NEXTflex Directional RNA Seq Kit (dUTP based and NEXTflex RNA-Seq Barcodes, Bioo Scientific, Austin, TX, USA). All libraries showed high quality with a distinct band at approximately 350 bp, checked with an Agilent High Sensitive DNA Chip on the 2100 Bioanalyzer and a concentration >10 nM. Concentration was measured using a Qubit 2.0 Fluorometer (Life Technologies). Sequencing was performed at the Max-Delbrück-Centrum (Berlin, Germany) on a HiSeq 2000 (Illumina, San Diego, CA, USA) to generate 100-bp paired end reads with a depth of 4–8 libraries per lane. The number of reads per library varied from 5,613,699 to 41,618,214 (mean 23,361,147). Read numbers were not significantly different between sexes (*F*_1,22_ = 1.417, *P* = 0.267) or species (*F*_1,22_ = 0.019, *P* = 0.892).

### Differential expression analysis

After sequencing, we determined if any of our candidate genes were differentially expressed between species or sexes using the Trinity differential expression pipeline^60^. Three biological replicates per sex per species (24 total) were used in the Trinity pipeline for differential expression analysis. For abundance estimation, reads from all samples were aligned against the subset of candidate transcripts from the *C. biguttulus* reference using bowtie^61^. Then, expression values were estimated using RSEM^62^. Differentially expressed transcripts were extracted using the DESeq2 algorithm^63^ with a trimmed mean of M-values normalization. Only contigs with an absolute value of log_2_ fold change >1 and a *P*-value < 0.05 were classified as differentially expressed and *P*-values were corrected for multiple testing^64^. We used counts as dependent variable and species and sex as explanatory variables with the interaction of species × sex. We compared the outcome of the DESeq2 package with the results of the egdeR^65^ algorithm. Both methods revealed identical differentially expressed contigs, although *P-*values differed. For the sake of clarity, results are shown only for the DESeq2 algorithm, because this algorithm is more conservative than the edgeR algorithm^66^.

### Coding sequence divergence analyses and estimation of substitution rates

In addition, we wanted to test whether our candidate FAS and ELO genes have undergone purifying or positive selection. To do this we estimated rates of nonsynonymous (dN) and synonymous (dS) substitutions between *C. biguttulus* and *C. mollis*. Based on the tblastn results of *C. biguttulus* and *C. mollis* (see Identification of FAS and ELO orthologs above) we calculated dN and dS substitutions for the FAS and ELO orthologs (Table 3) which we had identified before. Reads from all 12 *C. biguttulus* and 12 *C. mollis* (see differential expression analysis above) were pooled by species *in silico* then aligned to the *C. biguttulus* reference transcriptome (Mayer *et al*. unpublished). SNPs were called as described in Berdan *et al*.^43^ and used to create two “species-specific” transcriptomes using the FastaAlternateReferenceMaker from GATK^67^. We then used ‘transdecoder’ (part of the TRINITY package^59^) to determine Open Reading Frames (ORFs) and estimated dN/dS following the Yang & Nielsen approximate method^68^ implemented in KaKs_Calculator (Version 1.2)^69^.

### Phylogenetic analysis of fatty acid synthases and elongases

Finally, we wanted to examine FAS and ELO evolution at the level of class (insect). We reconstructed a phylogeny of the fatty acid synthase and elongase families. We first translated the nucleotide sequences of *C. biguttulus* and *C. mollis* into amino acid sequences using the translate tool server at http://www.expasy.org/tools/dna.html/. The open reading frames (ORFs) of these protein sequences were then used to extract homologs from selected representatives of all available insect orders from OrthoDB v8^70^, the NCBI database and AphidBase (http://www.aphidbase.com/aphidbase/) by a blastp search (see Supplementary Table S5 for selected species and Genbank accession numbers). The amino acid sequences were aligned using MAFFT version 7^71^. Prior to the phylogenetic analysis, poorly aligned positions were eliminated using GBlocks software^72^ with least stringent selection options. Phylogenetic analyses were conducted in PhylML 3.0^73^ with default settings. The domain structures of the FAS and elongase proteins were analyzed with MOTIF (http://www.genome.jp/tools/motif/).

### Data accessibility

The GC/MS data and the count matrix including the SRA accession number of grasshoppers are archived in dryad. Doi:10.5061/dryad.5qn13.

## Additional Information

**How to cite this article**: Finck, J. *et al*. Divergence of cuticular hydrocarbons in two sympatric grasshopper species and the evolution of fatty acid synthases and elongases across insects. *Sci. Rep.* **6**, 33695; doi: 10.1038/srep33695 (2016).

## Supplementary Material

Supplementary Information

## Figures and Tables

**Table 1:** Statistics of the cuticular hydrocarbon variation for adult male and female *Chorthippus biguttulus* and *C. mollis* grasshoppers.

Effect	PC1		PC2		PC3		PC4	
	F_3,121_	P	F_3,121_	P	F_3,121_	P	F_3,121_	P
Model	247.3	**<*****0.001***	21.9	**<*****0.001***	9.8	**<*****0.001***	5.3	***0.002***
Species	−6.31	**<*****0.001***	−0.53	0.315	2.00	**<*****0.001***	−1.01	***0.005***
Sex	0.58	0.078	−3.17	**<*****0.001***	0.77	***0.035***	−0.34	0.228
Species × Sex	−1.09	***0.042***	1.52	***0.029***	−3.07	**<*****0.001***	1.70	**<*****0.001***
**Tukey’s HSD** ***post-hoc*** **test**	**P**_**adj**_		**P**_**adj**_		**P**_**adj**_		**P**_**adj**_	
*C. mollis* F × *C. biguttulus* F	**<*****0.001***		0.745		**<*****0.001***		***0.024***	
*C. mollis* M × *C. biguttulus* M	**<*****0.001***		0.118		***0.028***		0.089	
*C. mollis* M × *C. biguttulus* F	**<*****0.001***		**<*****0.001***		0.852		0.598	
*C. mollis* F × *C. biguttulus* M	**<*****0.001***		**<*****0.001***		***0.044***		0.253	
*C. mollis* F × *C. mollis* M	0.615		***0.014***		**<*****0.001***		***0.001***	
*C. biguttulus* F × *C. biguttulus* M	0.288		**<*****0.001***		0.149		0.621	

Species, sex and the interaction between the two groups were tested using linear models for the principal component (PC) 1–5 with the PC scores as the dependent variable and species and sex as explanatory variables. Shown are the results for PC1-4 (model for PC5 showed no significance). Significant effects are indicated in bold and italics. Total n = 125.

**Table 2:** Factor loadings of each cuticular hydrocarbon peak on each of the five principal components (PC) in this study.

Peak	RI	Compound	PC1	PC2	PC3	PC4	PC5
1	2500	*n*-C25	0.14	**0.22**	0.19	0.08	0.05
2	2700	*n*-C27	−0.09	0.14	**0.27**	**0.25**	**0.28**
3	2900	*n*-C29	0.00	**0.25**	**0.43**	0.07	0.14
4	2975	3-MeC29	0.10	0.18	0.05	**−0.38**	−0.12
5	3100	*n*-C31	0.04	**0.21**	**0.46**	0.11	0.02
6	3133	13-MeC31	0.16	−0.11	−0.01	−0.09	0.11
7	3200	*n*-C32	−0.04	0.00	0.13	**0.20**	**−0.50**
8	3300	*n*-C33	−0.06	−0.01	**0.32**	0.11	−0.10
9	3332	11-/13-/15-MeC33	**0.20**	−0.04	−0.10	**0.22**	0.12
10	3357	unidentified	0.12	**−0.23**	0.09	−0.13	0.10
11	3360	15,19-/13,19-/11,21-diMeC33	**−0.26**	−0.03	0.01	0.04	0.03
12	3364	13,17-/13,19-/11,21-/9,19-diMeC33	**0.23**	0.10	−0.09	0.00	0.03
13	3382	13,17,21-/11,15,19-/9,15,23-diMeC33	**−0.22**	0.09	−0.07	0.04	−0.04
14	3432	10-/11-/12-/13-/14-MeC34	0.10	0.03	−0.18	0.01	**0.33**
15	3462	11,x-/12,x-/13,x-/14,x-diMeC34	**−0.23**	−0.04	−0.06	0.11	−0.06
16	3533	11-/13-/15-/17-MeC35	0.19	0.00	−0.13	**0.29**	0.14
17	3556	15,19-/13,17-/13,21-/11,21-diMeC35	**−0.27**	−0.02	0.01	−0.04	0.11
18	3561	13,17-/11,23-/9,21-diMeC35	0.19	**−0.27**	0.18	0.00	−0.09
19	3564	11,23-/9,21-diMeC35	0.10	**0.37**	−0.19	0.07	0.02
20	3776	11,19,23-/13,17,21-/13,17,23-triMeC35	**−0.27**	−0.02	0.02	−0.04	0.10
21	3581	13,17,21-/11,19,23-triMeC35	0.19	**−0.27**	0.19	0.00	−0.09
22	3583	11,19,23-/9,17,21-diMeC35	0.10	**0.37**	−0.16	0.06	−0.06
23	3607	3,x-diMeC35	−0.06	0.14	0.04	**−0.44**	**−0.40**
24	3632	12-/13-/14-/15-/16-MeC36	0.05	**−0.23**	**−0.26**	**0.29**	**−0.22**
25	3660	13,x-14,x-/15,x-diMeC36	**−0.23**	−0.07	−0.08	0.14	−0.06
26	3733	11-/13-/15-/17-/19-MeC37	**0.22**	0.10	0.07	0.01	0.05
27	3759	15,19-/15,21-/15,23-/13,23-diMeC37	**−0.26**	−0.03	−0.01	−0.01	0.12
28	3762	13,23-/11,23-/11,25-/9,23-/9,25-diMeC37	**0.26**	−0.01	−0.01	0.04	−0.09
29	3774	15,19,23-/13,17,23-/13,19,25-triMeC37	**−0.26**	−0.01	0.03	−0.06	0.10
30	3778	13,17,23-/11,19,25-/9,17,23-triMeC37	0.19	**−0.26**	0.17	0.01	−0.10
31	3780	11,19,25-/9,17,23-diMeC37	0.06	**0.27**	−0.10	−0.16	−0.13
32	3931	i-MeC39	0.08	−0.04	0.00	**−0.28**	**0.23**
33	3960	13,23-/13,25-diMeC39	0.06	−0.13	−0.04	**−0.34**	**0.29**
34	3963	11,23-/11,25-/9,23-/9,25-diMeC39	0.09	**0.21**	−0.19	0.10	−0.06

Loadings with absolute values >0.2 are indicated in bold.

**Table 3:** Overview of the ortholog assignment of the fatty acid synthase (FAS) and elongase families in *Chorthippus* grasshoppers.

Family	Cluster^a^	Contig name in reference transcriptome^b^	
		*C. biguttulus*	*C. mollis*
FAS	Cluster I	20030big_male-comp37496_c1_seq1	20003mol_P1-comp71695_c0_seq1
FAS	Cluster II-a	20013big_P1_male-comp38343_c0_seq2^c^	20016mol_P1_male-comp81435_c0_seq1
FAS	Cluster II-b	20011big_P1-comp52607_c0_seq1^c^	20003mol_P1-comp70825_c0_seq1
FAS	Cluster II-c	20011big_P1-comp58522_c0_seq1^c^	20003mol_P1-comp71027_c0_seq1
FAS	Cluster III	20030big_male-comp38169_c0_seq1	20164mol-comp17321_c0_seq1
Elo	baldspot	20010big_P1-comp55033_c0_seq1	20016mol_P1_male-comp83867_c0_seq1
Elo	Elo68	20013big_P1_male-comp131546_c0_seq1^d^	20015mol_P1_male-comp119420_c0_seq1
Elo	Elo68	20030big_male-comp106526_c0_seq1^d^	—
Elo	CG33110	20008big_male-comp98995_c0_seq1	20164mol-comp42127_c0_seq1
Elo	CG30008	20013big_P1_male-comp77836_c1_seq1^c^	20007mol_male-comp111352_c0_seq1
Elo	EloF	20030big_male-comp89598_c0_seq1^c^	20015mol_P1_male-comp86102_c0_seq1
Elo	james bond	—	20164mol-comp41288_c0_seq1
Elo	CG5278	20030big_male-comp88504_c2_seq1	20164mol-comp17390_c0_seq1
Elo	CG5326	20030big_male-comp94699_c0_seq1	20164mol-comp45532_c0_seq1
Elo		20010big_P1-comp54703_c0_seq1^e^	20056mol-comp120270_c0_seq1
Elo	CG31523	20030big_male-comp91260_c0_seq1	20056mol-comp120587_c6_seq3
Elo	CG31522	20008big_male-comp94799_c0_seq1	20164mol-comp39997_c0_seq1
Elo	CG2781	20030big_male-comp90320_c0_seq1	20007mol_male-comp113584_c0_seq1

^a^Compare Figs 2 and 3 for FAS and ELO clusters, respectively.

^b^See Supplementary Data for sequence information.

^c^No reciprocal best hit to the putative ortholog in *D. melanogaster.*

^d^Identical coding sequences.

^e^No ortholog in other insect orders.

**Table 4:** Overview of differentially expressed candidate genes^1^.

Class	Ortholog cluster	Species^2^		Sex^3^		Species × Sex	
		log2FC ± s.e.m.	P_adj_	log2FC ± s.e.m.	P_adj_	log2FC ± s.e.m.	P_adj_
FAS	Cluster II-a			3.08 ± 0.37	<0.001	−1.95 ± 0.46	<0.001
FAS	Cluster II-b	−1.52 ± 0.53	0.0347	−2.92±0.53	<0.001		
FAS	Cluster III			1.23 ± 0.31	<0.001		
ELO	CG16905 (EloF)			1.20 ± 0.30	<0.001		
ELO	CG30008			4.53 ± 0.49	<0.001		
ELO	CG5326			1.83 ± 0.34	<0.001		

^1^Extracted by the DESeq2 algorithm^61^.

^2^Negative values indicate higher expression in *C. biguttulus.*

^3^Positive and negative values indicate male- and female-biased expression, respectively.

## References

[b1] Chung H. & Carroll S. B. Wax, sex and the origin of species: Dual roles of insect cuticular hydrocarbons in adaptation and mating. Bioessays 37, 822–830 (2015). 25988392 10.1002/bies.201500014 PMC4683673

[b2] Singer T. L. Roles of hydrocarbons in the recognition systems of insects. Am. Zool. 38, 394–405 (1998).

[b3] Ferveur J. F. Cuticular hydrocarbons: their evolution and roles in *Drosophila* pheromonal communication. Behav. Genet. 35, 279–295 (2005). 15864443 10.1007/s10519-005-3220-5

[b4] Howard R. W. & Blomquist G. J. Ecological, behavioral, and biochemical aspects of insect hydrocarbons. Annu. Rev. Entomol. 50, 371–393 (2005). 15355247 10.1146/annurev.ento.50.071803.130359

[b5] Johansson B. G. & Jones T. M. The role of chemical communication in mate choice. Biol. Rev. 83, 265–289 (2007). 17437561 10.1111/j.1469-185X.2007.00009.x

[b6] Martin S. & Drijfhout F. A review of ant cuticular hydrocarbons. J. Chem. Ecol. 35, 1151–1161 (2009). 19866237 10.1007/s10886-009-9695-4

[b7] Geiselhardt S. F., Geiselhardt S. & Peschke K. Congruence of epicuticular hydrocarbons and tarsal secretions as a principle in beetles. Chemoecology 21, 181–186 (2011).

[b8] Howard R. W. Cuticular hydrocarbons and chemical communication in Insect Lipids: Chemistry, Biochemistry and Biology (eds. Stanley-Samuelson, D. W. & Nelson, ) 179–226 (University of Nebraska Press, 1993).

[b9] Bagnères A.-G. & Wicker-Thomas C. Chemical taxonomy with hydrocarbons in Insect Hydrocarbons: Biology, Biochemistry, and Chemical Ecology (Blomquist G. J. & Bagnères A.-G.) 121–162 (Cambridge University Press, 2010).

[b10] Blomquist G. J. Biosynthesis of cuticular hydrocarbons in Insect Hydrocarbons: Biology, Biochemistry, and Chemical Ecology (Blomquist G. J. & Bagnères A.-G.) 35–52 (Cambridge University Press, 2010).

[b11] Qiu Y. . An insect-specific P450 oxidative decarbonylase for cuticular hydrocarbon biosynthesis. Proc Natl. Acad. Sci. USA 109, 14858–14863 (2012). 22927409 10.1073/pnas.1208650109 PMC3443174

[b12] Chung H. . A single gene affects both ecological divergence and mate choice in *Drosophila*. Science 343, 1148–1151 (2014). 24526311 10.1126/science.1249998

[b13] Chirala S. S. & Wakil S. J. Structure and function of animal fatty acid synthase. Lipids 39, 1045–1053 (2004). 15726818 10.1007/s11745-004-1329-9

[b14] Smith S. & Tsai S.-C. The type I fatty acid and polyketide synthases: a tale of two megasynthases. Nat. Prod. Rep. 24, 1041–1072 (2007). 17898897 10.1039/b603600g PMC2263081

[b15] Hashimoto K. . The repertoire of desaturases and elongases reveals fatty acid variations in 56 eukaryotic genomes. J. Lipid Res. 49, 183–191 (2008). 17921532 10.1194/jlr.M700377-JLR200

[b16] Wagner A. The molecular origins of evolutionary innovations. Trends Genet. 27, 397–410 (2011). 21872964 10.1016/j.tig.2011.06.002

[b17] Gompel N., Prud’homme B., Wittkopp P. J., Kassner V. A. & Carroll S. B. Chance caught on the wing: *cis*-regulatory evolution and the origin of pigment patterns in *Drosophila*. Nature 433, 481–487 (2005). 15690032 10.1038/nature03235

[b18] Santos M. E. . The evolution of cichlid fish egg-spots is linked with a *cis*-regulatory change. Nature Comm. 5, 5149 (2014). 10.1038/ncomms6149 PMC4208096 25296686

[b19] Lynch M. & Conery J. S. The evolutionary fate and consequences of duplicate genes. Science 290, 1151–1155 (2000). 11073452 10.1126/science.290.5494.1151

[b20] Zhang J. Evolution by gene duplication: an update. Trends Ecol. Evol. 18, 292–298 (2003).

[b21] Hughes A. L. Gene duplication and the origin of novel proteins. Proc. Natl. Acad. Sci. USA 102, 8791–8792 (2005). 15956198 10.1073/pnas.0503922102 PMC1157061

[b22] Innan H. & Kondrashov F. The evolution of gene duplications: classifying and distinguishing between models. Nature Rev. Genet. 11, 97–108 (2010). 20051986 10.1038/nrg2689

[b23] Chen S., Krisnsky B. H. & Long M. New genes as drivers of phenotypic evolution. Nature Rev. Genet. 14, 645–660 (2013). 23949544 10.1038/nrg3521 PMC4236023

[b24] Nowak M. A., Boerlijst M. C., Cooke J. & Smith J. M. Evolution of genetic redundancy. Nature 388, 167–171 (1997). 9217155 10.1038/40618

[b25] Smadja C. & Butlin R. K. On the scent of speciation: the chemosensory system and its role in premating isolation. Heredity 102, 77–97 (2009). 18685572 10.1038/hdy.2008.55

[b26] Perdeck A. C. The isolating value of specific song patterns in two sibling species of grasshoppers (*Chorthippus brunneus* Thunb. and *C. biguttulus* L.). Behaviour 12, 1–75 (1958).

[b27] von Helversen D. & von Helversen O. Recognition of sex in the acoustic communication of the grasshopper *Chorthippus biguttulus* (Orthoptera, Acrididae). J. Comp. Physiol. A 180, 373–386 (1997).

[b28] Greenfield M. D. Acoustic Communication in Orthoptera in The bionomics of grasshoppers, Katydids and their kin (eds Gangwere S. K. .) 197–230 (CAB International, 1997).

[b29] Mayer F., Berger D., Gottsberger B. & Wolfram S. Non-ecological radiations in acoustically communicating grasshoppers? In Evolution in Action (ed Glaubrecht M.) 451–464 (Springer, 2010).

[b30] Ronacher B. & Stange N. Processing of acoustic signals in grasshoppers-A neuroethological approach towards female choice. J. Physiol.-Paris 107, 41–50 (2013). 22728472 10.1016/j.jphysparis.2012.05.005

[b31] Ritchie M. G. Are differences in song responsible for assortative mating between subspecies of the grasshopper *Chorthippus parallelus* (Orthoptera: Acrididae)? Anim. Behav. 39, 685–691 (1990).

[b32] Butlin R. K. What do hybrid zones in general, and the *Chorthippus parallelus* zone in particular, tell us about speciation in *Endless Forms: Species and Speciation* (eds Howard D. & Berlocher S. H.) 367–378 (Oxford University Press, 1998).

[b33] Simmons L. W. Pheromonal cues for the recognition of kin by female field crickets, Gryllus bimaculatus. Anim. Behav. 40, 192–195 (1990).

[b34] Tregenza T. & Wedell N. Definitive evidence for cuticular pheromones in a cricket. Anim. Behav. 54, 979–984 (1997). 9344449 10.1006/anbe.1997.0500

[b35] Mullen S. P., Mendelson T. C., Schal C. & Shaw K. L. Rapid evolution of cuticular hydrocarbons in a species radiation of acoustically diverse Hawaiian crickets (Gryllidae: Trigonidiinae: Laupala). Evolution 61, 223–231 (2007). 17300441 10.1111/j.1558-5646.2007.00019.x

[b36] Ryan K. M. & Sakaluk S. K. Dulling the senses: the role of the antennae in mate recognition, copulation and mate guarding in decorated crickets. Anim. Behav. 77, 1345–1350 (2009).

[b37] Thomas M. L. & Simmons L. W. Cuticular hydrocarbons influence female attractiveness to males in the Australian field cricket, *Teleogryllus oceanicus*. J. Evol. Biol. 23, 707–714 (2010). 20210834 10.1111/j.1420-9101.2010.01943.x

[b38] Grunshaw J. P. . Chemical taxonomic studies of cuticular hydrocarbons in locusts of the *Schistocerca americana* complex (Acrididae: Cyrtacanthacridinae): Chemical relationships between new world and old world species. J. Chem. Ecol. 16, 2835–2858 (1990). 24263258 10.1007/BF00979477

[b39] Lockey K. H. & Oraha V. S. Cuticular lipids of adult *Locusta migratoria migratoriodes* (R and F), *Schistocerca gregaria* (Forskål) (Acrididae) and other orthopteran species—II. Hydrocarbons. Comp. Biochem. Physiol. B 95, 721–744 (1990).

[b40] Chapman R. F., Espelie K. E. & Sword G. A. Use of cuticular lipids in grasshopper taxonomy: A study of variation in *Schistocerca shoshone* (Thomas). Biochem. Syst. Ecol. 23, 383–398 (1995).

[b41] Neems R. M. & Butlin R. K. Divergence in cuticular hydrocarbons between parapatric subspecies of the meadow grasshopper, *Chorthippus-parallelus* (Orthoptera, Acrididae). Biol. J. Linn. Soc. 54, 139–149 (1995).

[b42] Sutton B. D., Carlson D. A., Lockwood J. A. & Nunamaker R. A. Cuticular hydrocarbons of glacially-preserved *Melanoplus* (Orthoptera: Acrididae): identification and comparison with hydrocarbons of *M. sanguinipes* and *M. spretus*. J. Orthoptera Res. 5, 1–12 (1996).

[b43] Berdan E. L., Mazzoni C. J., Waurick I., Roehr J. T. & Mayer F. A population genomic scan in *Chorthippus* grasshoppers unveils previously unknown phenotypic divergence. Mol. Ecol. 24, 3918–3930 (2015). 26081018 10.1111/mec.13276

[b44] Dönitz J. . iBeetle-Base: a database for RNAi phenotypes in the red flour beetle *Tribolium castaneum*. Nucleic Acids Res. 43, D720–D725 (2014). 25378303 10.1093/nar/gku1054 PMC4383896

[b45] De Renobales M., Woodin T. S. & Blomquist G. J. *Drosophila melanogaster* fatty acid synthase. Characteristics and effect of protease inhibitors. Insect Biochem. 16, 887–894 (1986).

[b46] Juárez M. P., Ayala S. & Brenner R. R. Methyl-branched fatty acid biosynthesis in *Triatoma infestans*. Insect Biochem. Mol. 26, 593–598 (1996).

[b47] Blomquist G. J. . Methyl-branched fatty acids and their biosynthesis in the house fly, *Musca domestica* L. (Diptera: Muscidae). Insect Biochem. Mol. 24, 803–810 (1994).

[b48] Buckner J. S., Kolattukudy P. E. & Rogers L. Synthesis of multimethyl-branched fatty acids by avian and mammalian fatty acid synthetase and its regulation by malonyl-CoA decarboxylase in the uropygial gland. Arch. Biochem. Biophys. 186, 152–163 (1978). 629531 10.1016/0003-9861(78)90474-5

[b49] Kolattukudy P. E., Rogers L. M. & Balapangu A. Synthesis of methyl-branched fatty acids from methylmalonyl-CoA by fatty acid synthase from both the liver and the harderian gland of the guinea pig. Arch. Biochem. Biophys. 255, 205–209 (1987). 3592662 10.1016/0003-9861(87)90312-2

[b50] Falcón T. . Exoskeleton formation in *Apis mellifera*: Cuticular hydrocarbons profiles and expression of desaturase and elongase genes during pupal and adult development. Insect Biochem. Mol. Biol. 50, 68–81 (2014). 24813723 10.1016/j.ibmb.2014.04.006

[b51] Chertemps T. . A female-biased expressed elongase involved in long-chain hydrocarbon biosynthesis and courtship behavior in *Drosophila melanogaster*. Proc. Natl. Acad. Sci. USA 104, 4273–4278 (2007). 17360514 10.1073/pnas.0608142104 PMC1838592

[b52] Wicker-Thomas C. & Chertemps T. Molecular biology and genetics of hydrocarbon production in Insect Hydrocarbons: Biology, Biochemistry, and Chemical Ecology (Blomquist G. J. & Bagnères A.-G.) 53–74 (Cambridge University Press, 2010).

[b53] Dembeck L. M. . Genetic architecture of natural variation in cuticular hydrocarbon composition in *Drosophila melanogaster*. eLife 4, e09861 (2015) 26568309 10.7554/eLife.09861 PMC4749392

[b54] Carlson D. A., Bernier U. R. & Sutton B. D. Elution patterns from capillary GC for methyl-branched alkanes. J. Chem. Ecol. 24, 1845–1865 (1998).

[b55] Aitchison J. The Statistical Analysis of Compositional Data (Chapman and Hall, 1986).

[b56] Geiselhardt S., Otte T. & Hilker M. Looking for a similar partner: host plants shape mating preferences of herbivorous insects by altering their contact pheromones. Ecol. Lett. 15, 971–977 (2012). 22708843 10.1111/j.1461-0248.2012.01816.x

[b57] Lê S., Josse J. & Husson F. FactoMineR : An R package for multivariate analysis. J. Stat. Softw. 25, 1–18 (2008).

[b58] R Core Team. R: A Language and Environment for Statistical Computing. *R Foundation for Statistical Computing.* Vienna, Austria. Available at: www.R-project.org. (2013).

[b59] Tatusov R. L., Koonin E. V. & Lipma D. J. A genomic perspective on protein families. Science 278, 631–637 (1997). 9381173 10.1126/science.278.5338.631

[b60] Haas B. J. . De novo transcript sequence reconstruction from RNA-seq using the Trinity platform for reference generation and analysis. Nat. Protoc. 8, 1494–1512 (2013). 23845962 10.1038/nprot.2013.084 PMC3875132

[b61] Langmead B., Trapnell C., Pop M. & Salzberg S. L. Ultrafast and memory-efficient alignment of short DNA sequences to the human genome. Genome Biol. 10, R25 (2009). 19261174 10.1186/gb-2009-10-3-r25 PMC2690996

[b62] Li B. & Dewey C. N. RSEM: accurate transcript quantification from RNA-Seq data with or without a reference genome. BMC Bioinformatics 12, 323 (2011). 21816040 10.1186/1471-2105-12-323 PMC3163565

[b63] Love M. I., Huber W. & Anders S. Moderated estimation of fold change and dispersion for RNA-seq data with DESeq2. Genome Biol. 15, 550 (2014). 25516281 10.1186/s13059-014-0550-8 PMC4302049

[b64] Benjamini Y. & Hochberg Y. Controlling the false discovery rate: a practical and powerful approach to multiple testing. J. R. Stat. Soc. B 57, 289–300 (1995).

[b65] Robinson M. D., McCarthy D. J. & Smyth G. K. edgeR: A Bioconductor package for differential expression analysis of digital gene expression data. Bioinformatics 26, 139–140 (2009). 19910308 10.1093/bioinformatics/btp616 PMC2796818

[b66] Robles J. A. . Efficient experimental design and analysis strategies for the detection of differential expression using RNA-Sequencing. BMC Genomics 13, 484 (2012). 22985019 10.1186/1471-2164-13-484 PMC3560154

[b67] McKenna A. . The Genome Analysis Toolkit: a MapReduce framework for analyzing next-generation DNA sequencing data. Genome Res. 20, 1297–1303 (2010). 20644199 10.1101/gr.107524.110 PMC2928508

[b68] Yang Z. & Nielsen R. Estimating synonymous and nonsynonymous substitution rates. Mol. Biol. Evol. 13, 105–114 (1996). 8583885 10.1093/oxfordjournals.molbev.a025549

[b69] Zhang Z. . KaKs_Calculator: calculating Ka and Ks through model selection and model averaging. Genomics Proteomics Bioinforma. 4, 259–263 (2006). 10.1016/S1672-0229(07)60007-2 PMC5054075 17531802

[b70] Kriventseva E. V. . OrthoDB v8: update of the hierarchical catalog of orthologs and the underlying free software. Nucleic Acids Res. 43, D250–D256 (2014). 25428351 10.1093/nar/gku1220 PMC4383991

[b71] Katoh K. & Standley D. M. MAFFT Multiple Sequence Alignment Software Version 7: Improvements in performance and usability. Mol. Biol. Evol. 30, 772–780 (2013). 23329690 10.1093/molbev/mst010 PMC3603318

[b72] Talavera G. & Castresana J. Improvement of phylogenies after removing divergent and ambiguously aligned blocks from protein sequence alignments. Syst. Biol. 56, 564–577 (2007). 17654362 10.1080/10635150701472164

[b73] Guindon S. . New algorithms and methods to estimate maximum-likelihood phylogenies: Assessing the performance of PhyML 3.0. Syst. Biol. 59, 307–321 (2010). 20525638 10.1093/sysbio/syq010

