# Supplementary material for: Divergence of cuticular hydrocarbons in two sympatric grasshopper species and the evolution of fatty acid synthases and elongases across insects
Source: Sci Rep. 2016 Sep 28;6:33695. doi: 10.1038/srep33695 (PMC5039406; doi:10.1038/srep33695)
Supplement: Supplementary Information [file srep33695-s1.pdf]

Supplementary Information for:

Divergence of cuticular hydrocarbons in two sympatric grasshopper species and the evolution of fatty acid synthases and elongases across insects

Jonas Finck*; Emma L. Berdan; Frieder Mayer; Bernhard Ronacher; Sven Geiselhardt

*To whom correspondence should be addressed

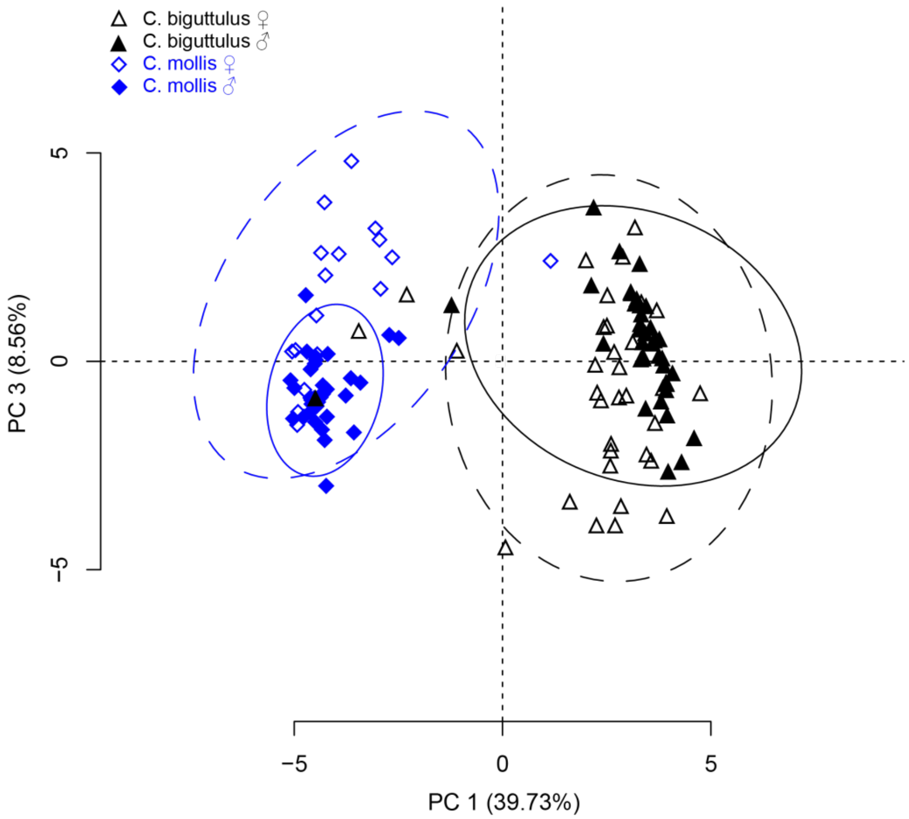

Figure S1. Principal component analysis (PCA) of cuticular hydrocarbon (CHC) phenotypes of male and female *Chorthippus biguttulus* and *Chorthippus mollis*. Shown are principal component (PC) 1 *versus* 3 with variances explained by each PC given in parentheses. Ellipses indicate 95% confidence intervals. The PCA is based on the relative composition of 34 CHC peaks (see Table 1 for loadings).

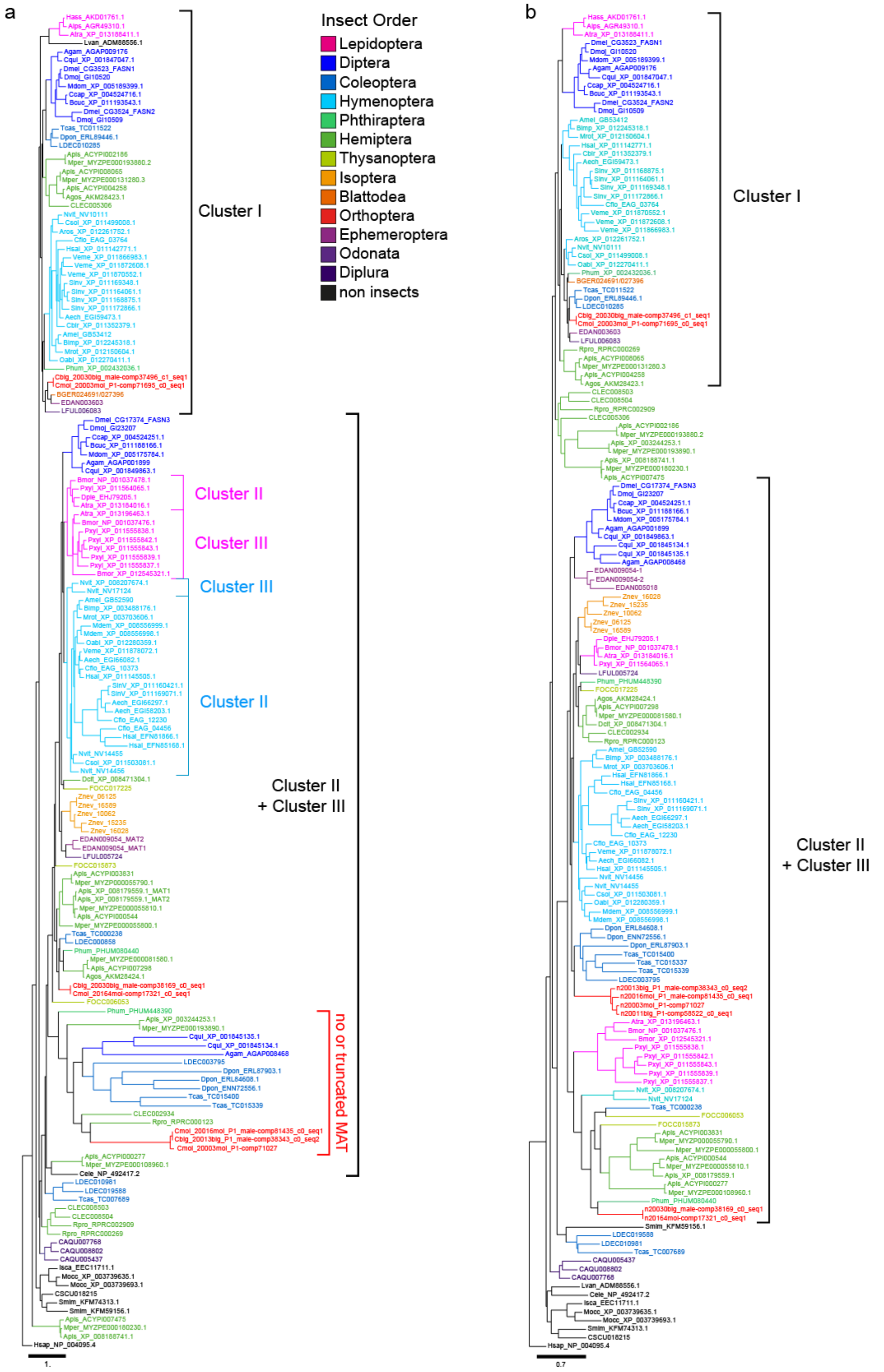

Figure S2. Phylogenetic relationship of fatty acid synthases in insects based on (a) the MAT and (b) the KS domain, respectively. The maximum-likelihood tree was computed based on 157 protein sequences from 44 insect species of 13 insect orders and seven non-insects outgroup species from three different phyla. The tree is rooted with the human FAS. The four letters code indicate the species, followed by the Genbank accession numbers (see Supplementary Table S5 for details).

Table S1. Mean (± S.D.) relative composition (%) of the cuticular hydrocarbon profiles of *Chorthippus biguttulus* and *C. mollis* grasshoppers

Peak	Retention index	Compound	*C. mollis*		*C. biguttulus*	
			Females N = 17	Males N =34	Females N = 40	Males N = 34
1	2500	*n*-C25	2.6±1.4	1.7±1.0	5.3±2.8	4.1±3.0
2	2700	*n*-C27	12.2±4.3	13.3±4.3	8.6±3.7	8.4±3.7
3	2900	*n*-C29	26.4±6.3	19.1±3.2	21.3±3.8	18.0±4.0
4	2975	3-MeC29	0.1±0.3	-	0.3±0.3	0.1±0.2
5	3100	*n*-C31	10.0±2.4	6.5±1.3	8.5±2.0	7.5±2.4
6	3133	13-MeC31	-	*tr*	0.2±0.3	0.2±0.2
7	3200	*n*-C32	*tr*	*tr*	*tr*	-
8	3300	*n*-C33	1.3±0.4	0.9±0.3	0.8±0.5	0.9±0.5
9	3332	11-/13-/15-MeC33	*tr*	0.3±0.3	1.7±1.2	1.4±0.8
10	3357	unidentified	-	-	-	0.8±0.8
11	3360	15,19-/13,19-/11,21-diMeC33	3.3±1.6	4.7±1.0	0.2±0.7	0.2±1.1
12	3364	13,17-/13,19-/11,21-/9,19-diMeC33	0.2±0.8		4.0±3.7	1.8±1.5
13	3382	13,17,21-/11,15,19-/9,15,23-diMeC33	2.2±1.0	2.2±0.5	0.6±1.4	0.2±0.7
14	3432	10-/11-/12-/13-/14-MeC34	-	-	0.2±0.4	0.1±0.3
15	3462	11,x-/12,x-/13,x-/14,x-diMeC34	0.6±0.6	0.7±0.3	*tr*	*tr*
16	3533	11-/13-/15-/17-MeC35	1.4±0.7	2.3±0.7	5.7±1.9	5.6±1.9
17	3556	15,19-/13,17-/13,21-/11,21-diMeC35	13.2±5.1	22.8±2.9	1.7±5.2	1.4±5.8
18	3561	13,17-/11,23-/9,21-diMeC35	-	-	8.3±11.0	24.9±11.5
19	3564	11,23-/9,21-diMeC35	0.5±2.1	-	9.0±8.8	1.1±3.7
20	3776	11,19,23-/13,17,21-/13,17,23-triMeC35	11.8±4.5	12.5±2.0	0.8±2.6	0.7±2.9
21	3581	13,17,21-/11,19,23-triMeC35	-	-	1.9±2.8	5.0±2.5
22	3583	11,19,23-/9,17,21-diMeC35	0.2±0.9	-	2.0±2.5	0.2±0.6
23	3607	3,x-diMeC35	1.1±1.8	0.1±0.3	0.2±0.6	-
24	3632	12-/13-/14-/15-/16-MeC36	0.4±0.6	1.3±0.4	1.2±0.7	1.5±0.6
25	3660	13,x-14,x-/15,x-diMeC36	0.6±0.6	0.8±0.4	*tr*	*tr*
26	3733	11-/13-/15-/17-/19-MeC37	1.2±0.3	1.2±0.4	2.7±1.0	2.5±0.9
27	3759	15,19-/15,21-/15,23-/13,23-diMeC37	4.0±1.7	6.0±1.5	0.7±2.2	0.4±1.8
28	3762	13,23-/11,23-/11,25-/9,23-/9,25-diMeC37	0.2±1.0	-	8.0±3.0	8.2±3.4
29	3774	15,19,23-/13,17,23-/13,19,25-triMeC37	4.1±2.0	3.3±1.0	0.4±1.1	0.2±0.9
30	3778	13,17,23-/11,19,25-/9,17,23-triMeC37	-	-	0.9±1.4	1.7±0.9
31	3780	11,19,25-/9,17,23-diMeC37	-	-	0.3±0.8	0.3±1.1
32	3931	i-MeC39	-	-	*tr*	0.1±0.2
33	3960	13,23-/13,25-diMeC39	*tr*	-	0.1±0.3	0.2±0.5
34	3963	11,23-/11,25-/9,23-/9,25-diMeC39	-	-	0.8±1.2	0.1±0.4
		Number of compounds	16.1±1.9	16.9±1.1	16.7±1.8	16.9±1.6
		n-alkanes	52.7±9.9	41.6±7.1	44.6±7.6	39.0±10.1
		methyl-branched alkanes	3.0±1.2	5.1±1.5	11.5±3.8	11.3±3.7
		dimethyl-branched alkanes	22.7±6.3	35.1±4.6	32.8±5.5	38.4±8.5
		trimethyl-branched alkanes	18.3±6.4	17.9±2.9	6.5±4.0	8.0±3.6
		others	0.4±0.3	0.1±0.1	3.4±2.0	2.8±1.4

tr traces (<0.1%)

Table S2. Mean (S.D.) ratios of peak heights of fragment ions m/z 140, m/z 168, m/z 196, and m/z 224 diagnostic for methyl-branched alkanes with first methyl-branch at position 9, 11, 13, and 15, respectively. The two most prominent fragment ions of each peak are indicated in bold.

Compound	Retention index				*C.biguttulus*								*C. mollis*							
					Females				Males				Females				Males			
	140	168	196	224	140	168	196	224	140	168	196	224	140	168	196	224	140	168	196	224
MeC31	3140	3136	3132	-	**37** (20)	15 (7)	**48** (23)	-	14 (17)	20 (14)	**66** (21)	-	-	-	-	-	-	-	-	-
MeC33	3335	3332	3329	3327	**35** (24)	21 (11)	**32** (15)	13 (11)	8 (11)	18 (9)	**40** (11)	**34** (12)	10 (11)	24 (14)	**34** (10)	**35** (19)	2 (7)	16 (12)	**37** (10)	**44** (13)
diMeC33	3364	3362	3358	3354	**40** (17)	**39** (12)	16 (17)	5 (12)	21 (10)	**36** (10)	**37** (14)	7 (14)	6 (10)	8 (6)	**32** (7)	**54** (14)	3 (2)	11 (12)	**41** (10)	**45** (13)
triMeC33	3386	3381	3376	-	**54** (26)	**32** (17)	14 (20)	-	**46** (20)	**45** (15)	9 (18)	-	17 (13)	**46** (9)	**38** (9)	-	12 (4)	**34** (10)	**34** (11)	-
MeC35	3535	3533	3530	3529	14 (8)	**34** (9)	**37** (10)	15 (8)	3 (5)	**24** (7)	**49** (7)	**24** (6)	11 (11)	24 (8)	**41** (9)	**24** (9)	0 (2)	20 (10)	**46** (7)	**34** (8)
diMeC35	3565	3562	3558	3555	27 (17)	**34** (10)	**35** (22)	5 (10)	4 (10)	**16** (14)	**78** (23)	3 (12)	5 (12)	16 (5)	**40** (7)	**40** (10)	0 (1)	9 (5)	**46** (6)	**45** (5)
triMeC35	3585	3582	3576	-	**36** (24)	**36** (11)	28 (29)	-	4 (11)	**39** (10)	**57** (13)	-	4 (15)	20 (7)	**77** (13)	-	-	16 (6)	**84** (6)	-
MeC37	3735	3733	3730	3728	3 (5)	**26** (8)	**52** (11)	19 (5)	0 (1)	**20** (8)	**63** (9)	16 (7)	1 (4)	15 (9)	**44** (8)	**39** (9)	-	6 (7)	**48** (8)	**46** (8)
diMeC37	3765	3762	3760	3754	22 (13)	**30** (13)	**44** (21)	3 (9)	3 (9)	**12** (16)	**83** (24)	3 (11)	3 (9)	12 (11)	**50** (11)	**36** (11)	-	3 (5)	**53** (7)	**44** (5)
triMeC37	3785	3780	3775	3771	**31** (25)	**25** (18)	**25** (31)	9 (19)	3 (12)	**5** (12)	**90** (21)	1 (8)	4 (17)	3 (8)	**38** (11)	**55** (16)	-	-	**43** (7)	**57** (7)

Table S3. Coding sequences analysis. Calculation of substitution rates of FAS and ELO candidate genes

Family	Contig name in reference transcriptome of *C. biguttulus*	Length[a]	Substitutions			dN/dS	*P*
			N	S	total		
FAS	20030big_male-comp37496_c1_seq1	7365	17	64	81	0.109	<0.0001
FAS	20013big_P1_male-comp38343_c0_seq2	6936	1	3	4	0.103	0.0413
FAS	20011big_P1-comp52607_c0_seq1	1149	3	0	3	-	-
FAS	20030big_male-comp38169_c0_seq1	6531	16	39	55	0.102	<0.0001
Elo	20010big_P1-comp55033_c0_seq1	870	-	2	2	-	-
Elo	20013big_P1_male-comp131546_c0_seq1[b]	870	0	0	0	-	-
Elo	20030big_male-comp106526_c0_seq1[b]	747	0	0	0	-	-
Elo	20008big_male-comp98995_c0_seq1	1209	1	2	3	0.129	0.1038
Elo	20013big_P1_male-comp77836_c1_seq1	810	2	9	11	0.003	<0.0001
Elo	20030big_male-comp89598_c0_seq1	795	1	6	7	0.061	0.002
Elo	20030big_male-comp88504_c2_seq1	948	1	12	13	0.028	0.0668
Elo	20030big_male-comp94699_c0_seq1	303	3	0	3	-	-
Elo	20010big_P1-comp54703_c0_seq1	1107	3	10	13	0.051	0.0891
Elo	20030big_male-comp91260_c0_seq1	1005	1	5	6	0.046	0.0011
Elo	20008big_male-comp94799_c0_seq1	963	0	2	2	0	-
Elo	20030big_male-comp90320_c0_seq1	954	0	5	5	0	-

[a] Length of the open reading frame of *C. biguttulus.*

[b] Identical coding sequences.

Table S4. Grasshoppers used for genetic analysis.

Species	Sex	Population	Storage	Date
C. biguttulus	F	Alterlangen	liquid nitrogen	8/22/2013
C. biguttulus	F	Alterlangen	liquid nitrogen	8/22/2013
C. biguttulus	F	Alterlangen	liquid nitrogen	8/22/2013
C. biguttulus	F	Berlin	liquid nitrogen	9/16/2012
C. biguttulus	F	Berlin	RNA later	9/16/2013
C. biguttulus	F	Berlin	RNA later	9/27/2013
C. biguttulus	M	Alterlangen	liquid nitrogen	8/16/2013
C. biguttulus	M	Alterlangen	liquid nitrogen	8/16/2013
C. biguttulus	M	Alterlangen	liquid nitrogen	8/16/2013
C. biguttulus	M	Berlin	RNA later	9/9/2013
C. biguttulus	M	Berlin	RNA later	9/12/2013
C. biguttulus	M	Berlin	RNA later	9/30/2013
C. mollis	F	Alterlangen	liquid nitrogen	8/27/2013
C. mollis	F	Alterlangen	liquid nitrogen	8/27/2013
C. mollis	F	Alterlangen	liquid nitrogen	8/27/2013
C. mollis	F	Berlin	RNA later	9/12/2013
C. mollis	F	Berlin	RNA later	9/27/2013
C. mollis	F	Berlin	liquid nitrogen	9/28/2012
C. mollis	M	Alterlangen	liquid nitrogen	8/16/2013
C. mollis	M	Alterlangen	liquid nitrogen	8/16/2013
C. mollis	M	Alterlangen	liquid nitrogen	8/16/2013
C. mollis	M	Berlin	liquid nitrogen	9/28/2012
C. mollis	M	Berlin	liquid nitrogen	9/10/2012
C. mollis	M	Berlin	liquid nitrogen	9/17/2012

Table S5. List of fatty acid synthases and elongases used in phylogenetic analyses.

Order	Taxon	Code	Accession number, ensemble identifier	Database
**Fatty acid synthases**				
Chordata	*Homo sapiens*	Hsap	NP_004095.4	NCBI
Nematoda	*Caenorhabditis elegans*	Cele	NP_492417.2	NCBI
Chelicerata	*Stegodyphus mimosarum*	Smim	KFM74313.1	NCBI
			KFM59156.1	NCBI
	Centruroides exilicauda	Cexi	CSCU18215	i5k pilot project
	Metaseiulus occidentalis	Mocc	XP_003739693.1	NCBI
			XP_003739635.1	NCBI
	Ixodes scapularis	Isca	EEC11711.1	NCBI
Crustacea	*Litopenaeus vannamei*	Lvan	ADM88556.1	NCBI
Diplura	*Catajapyx aquilonaris*	Caqu	CAQU005437	i5k pilot project
			CAQU007768	i5k pilot project
			CAQU008802	i5k pilot project
Odonata	*Ladona fulva*	Lful	LFUL005724	i5k pilot project
			LFUL006083	i5k pilot project
Ephemeroptera	*Ephemera danica*	Edan	EDAN005018	i5k pilot project
			EDAN009054	i5k pilot project
			EDAN003603	i5k pilot project
Orthoptera	*Chorthippus biguttulus*	Cbig	20011big_P1-comp52607_c0_seq1	present study
			20011big_P1-comp58522_c0_seq1	present study
			20013big_P1_male-comp38343_c0_seq2	present study
			20030big_male-comp17321_c0_seq1	present study
			20030big_male-comp37496_c1_seq1	present study
	Chorthippus mollis	Cmol	20003mol_P1-comp70825_c0_seq1	present study
			20003mol_P1-comp71027_c0_seq1	present study
			20003mol_P1-comp71695_c0_seq1	present study
			20016mol_P1_male-comp81435_c0_seq1	present study
			20164mol-comp17321_c0_seq1	present study
	Stenobothrus lineatus	Slin	GAUZ02046098	NCBI
			GAUZ02048699	NCBI
			GAUZ02046522	NCBI
			GAUZ02049228	NCBI
			GAUZ02048496	NCBI
Blattodea	*Blattella germanica*	Bger	GBID01000802	NCBI
			GBID01001122	NCBI
			GBID01002181	NCBI
Isoptera	*Zootermopsis nevadensis*	Znev	Znev_06125	termitegenome.org
			Znev_10062	termitegenome.org
			Znev_15235	termitegenome.org
			Znev_16028	termitegenome.org
			Znev_16589	termitegenome.org
Phthiraptera	*Pediculus humanus*	Phum	PHUM565830	NCBI
			PHUM448390	NCBI
			PHUM080440	OrthoDB v8
Hemiptera	*Aphis gossypii*	Agos	AKM28423.1	NCBI
			AKM28424.1	NCBI
	Acyrthosiphon pisum	Apis	ACYPI000277	AphidBase
			ACYPI000544	AphidBase
			ACYPI002186	AphidBase
			ACYPI003831	AphidBase
			ACYPI004258	AphidBase
			ACYPI007298	AphidBase
			ACYPI007475	AphidBase
			ACYPI008065	AphidBase
			XP_003244253.1	NCBI
			XP_008188741.1	NCBI
			XP_001945287.2	NCBI
	Myzus persicae	Mper	MYZPE13164_G006_v1.0_000131280.3	AphidBase
			MYZPE13164_G006_v1.0_000055800.1	AphidBase
			MYZPE13164_G006_v1.0_000055790.1	AphidBase
			MYZPE13164_G006_v1.0_000055810.1	AphidBase
			MYZPE13164_G006_v1.0_000180230.1	AphidBase
			MYZPE13164_G006_v1.0_000193890.1	AphidBase
			MYZPE13164_G006_v1.0_000193880.2	AphidBase
			MYZPE13164_G006_v1.0_000108960.1	AphidBase
			MYZPE13164_G006_v1.0_000081580.1	AphidBase
	Diaphorina citri	Dcit	XP_008471304.1	NCBI
	Cimex lectularius	Clec	CLEC002934	i5k pilot project
			CLEC005306	i5k pilot project
			CLEC006503	i5k pilot project

			CLEC008504	i5k pilot project
	Rhodnius prolixus	Rpro	RPRC000269	OrthoDB v8
			RPRC002909	OrthoDB v8
			RPRC000123	OrthoDB v8
Thysanoptera	*Frankliniella occidentalis*	Focc	FOCC015873	i5k pilot project
			FOCC017225	i5k pilot project
			FOCC006053	i5k pilot project
Coleoptera	*Dendroctonus ponderosae*	Dpon	ERL89446.1	NCBI
			ERL87903.1	NCBI
			ENN72556.1	NCBI
			ERL84608.1	NCBI
	Leptinotarsa decemlineata	Ldec	LDEC000858	i5k pilot project
			LDEC001692	i5k pilot project
			LDEC003795	i5k pilot project
			LDEC010285	i5k pilot project
			LDEC010981	i5k pilot project
			LDEC019588	i5k pilot project
			LEDC003795	i5k pilot project
	Tribolium castaneum	Tcas	TC011522	BeetleBase
			TC000238	BeetleBase
			TC015337	BeetleBase
			TC015339	BeetleBase
			TC015400	BeetleBase
			TC007689	BeetleBase
Hymenoptera	*Athalia rosae*	Aros	XP_012261752.1	NCBI
	Acromyrmex echinator	Aech	EGI66297.1	NCBI
			EGI66082.1	NCBI
			EGI59473.1	NCBI
			EGI58203.1	NCBI
	Apis mellifera	Amel	GB53412	BeeBase
			GB52590	NCBI
	Bombus impatiens	Bimp	XP_012245318.1	NCBI
			XP_003488176.1	NCBI
	Cerapachys biroi	Cbir	XP_011352379.1	NCBI
	Camponotus floridanus	Cflo	XP_011264667.1	NCBI
			XP_011256122.1	NCBI
	Ceratosolen solmsi marchali	Csol	XP_011503081.1	NCBI
			XP_011499008.1	NCBI
	Harpegnathos saltator	Hsal	XP_011142771.1	NCBI
			XP_011145505.1	NCBI
			EFN81866.1	NCBI
			EFN85168.1	NCBI
	Megachile rotundata	Mrot	XP_012150604.1	NCBI
			XP_003703606.1	NCBI
	Nasionia vitripennis	Nvit	NV10111	OrthoDB v8
			NV14455	OrthoDB v8
			NV14456	OrthoDB v8
			NV17124	OrthoDB v8
			XP_008207674.1	NCBI
	Orussus abietinus	Oabi	XP_012270411.1	NCBI
			XP_012280359.1	NCBI
	Solenopsis invicta	Sinv	XP_011160421.1	NCBI
			XP_011164061.1	NCBI
			XP_011168875.1	NCBI
			XP_011169071.1	NCBI
			XP_011169348.1	NCBI
			XP_011172866.1	NCBI
	Vollenhovia emeryi	Veme	XP_011866983.1	NCBI
			XP_011870552.1	NCBI
			XP_011872608.1	NCBI
			XP_011878072.1	NCBI
Diptera	*Anopheles gambiae*	Agam	AGAP001899	VectorBase
			AGAP008468	VectorBase
			AGAP009176	VectorBase
	Bactrocera cucurbitae	Bcuc	XP_011188166.1	NCBI
			XP_011193543.1	NCBI
	Ceratitis capitata	Ccap	XP_004524716.1	NCBI
			XP_004524251.1	NCBI
	Culex quinquefasciatus	Cqui	CPIJ003494	VectorBase
			CPIJ003495	VectorBase
			CPIJ005595	VectorBase
			CPIJ008367	VectorBase
	Drosophila melanogaster	Dmel	CG3523	FlyBase

			CG3524	FlyBase
			CG17374	FlyBase
	Drosophila mojavensis	Dmoj	GI10509	FlyBase
			GI10520	FlyBase
			GI23207	FlyBase
	Musca domestica	Mdom	XP_005175784.1	NCBI
			XP_005189399.1	NCBI
Lepidoptera	*Agrotis ipsilon*	Aips	AGR49310.1	NCBI
	Amyelois transitella	Atra	XP_013188411.1	NCBI
			XP_013184016.1	NCBI
	Bombyx mori	Bmor	NP_001037478.1	NCBI
			NP_001037476.1	NCBI
			XP_012545321.1	NCBI
	Danaus plexippus	Dple	DPOGS115634	MonarchBase
	Helicoverpa assulta	Hass	AKD01761.1	NCBI
	Plutella xylostella	Pxyl	XP_011564065.1	NCBI
			XP_011555838.1	NCBI
			XP_011555842.1	NCBI
			XP_011555843.1	NCBI
			XP_011555839.1	NCBI
			XP_011555837.1	NCBI
			Elongases	
Chordata	*Bos taurus*	Btau	ELOVL1	NCBI
			ELOVL5	NCBI
			ELOVL7	NCBI
			ELOVL2	NCBI
			ELOVL4	NCBI
			ELOVL6	NCBI
			ELOVL3	NCBI
Crustacea	*Daphnia pulex*	Dpul	DAPPUDRAFT_260977	JGI Genome Portal
			DAPPUDRAFT_309034	JGI Genome Portal
			DAPPUDRAFT_108808	JGI Genome Portal
			DAPPUDRAFT_128564	JGI Genome Portal
			DAPPUDRAFT_307239	JGI Genome Portal
			DAPPUDRAFT_307269	JGI Genome Portal
			DAPPUDRAFT_307270	JGI Genome Portal
			DAPPUDRAFT_322487	JGI Genome Portal
			DAPPUDRAFT_324294	JGI Genome Portal
			DAPPUDRAFT_324296	JGI Genome Portal
			DAPPUDRAFT_41014	JGI Genome Portal
			DAPPUDRAFT_41788	JGI Genome Portal
Odonata	Ladona fulva	Lful	LFUL001095	i5k pilot project
			LFUL002423	i5k pilot project
			LFUL006533	i5k pilot project
			LFUL007078	i5k pilot project
			LFUL012359	i5k pilot project
			LFUL012361	i5k pilot project
			LFUL012474	i5k pilot project
			LFUL013814	i5k pilot project
			LFUL013815	i5k pilot project
			LFUL013816	i5k pilot project
			LFUL013817	i5k pilot project
			LFUL013818	i5k pilot project
Ephemeroptera	Ephemera danica	Edan	EDAN004593	i5k pilot project
			EDAN004594	i5k pilot project
			EDAN004595	i5k pilot project
			EDAN005619	i5k pilot project
			EDAN006119	i5k pilot project
			EDAN006220	i5k pilot project
			EDAN015111	i5k pilot project
			EDAN015113	i5k pilot project
			EDAN015114	i5k pilot project
			EDAN015384	i5k pilot project
			EDAN015575	i5k pilot project
			EDAN018111	i5k pilot project
Orthoptera	*Chorthippus biguttulus*	Cbig	20001big_P1-comp67133_c0_seq1	present study
			20008big_male-comp94799_c0_seq1	present study
			20008big_male-comp98995_c0_seq1	present study
			20010big_P1-comp54703_c0_seq1	present study
			20010big_P1-comp55033_c0_seq1	present study
			20013big_P1_male-comp131546_c0_seq1	present study
			20013big_P1_male-comp77836_c1_seq1	present study
			20030big_male-comp106526_c0_seq1	present study

			20030big_male-comp88504_c2_seq1	present study
			20030big_male-comp89598_c0_seq1	present study
			20030big_male-comp90320_c0_seq1	present study
			20030big_male-comp91260_c0_seq1	present study
			20030big_male-comp94699_c0_seq1	present study
	Chorthippus mollis	Cmol	20007mol_male-comp111352_c0_seq1	present study
			20007mol_male-comp113584_c0_seq1	present study
			20015mol_P1_male-comp119420_c0_seq1	present study
			20015mol_P1_male-comp86102_c0_seq1	present study
			20016mol_P1_male-comp83867_c0_seq1	present study
			20056mol-comp120270_c0_seq1	present study
			20056mol-comp120587_c6_seq3	present study
			20164mol-comp17390_c0_seq1	present study
			20164mol-comp39997_c0_seq1	present study
			20164mol-comp41288_c0_seq1	present study
			20164mol-comp42127_c0_seq1	present study
			20164mol-comp45532_c0_seq1	present study
Isoptera	*Zootermopsis nevadensis*	Znev	KDR06850.1	termitegenome.org
			KDR06852.1	termitegenome.org
			KDR11198.1	termitegenome.org
			KDR12083.1	termitegenome.org
			KDR19399.1	termitegenome.org
			KDR19403.1	termitegenome.org
			KDR19404.1	termitegenome.org
			KDR19452.1	termitegenome.org
			KDR20355.1	termitegenome.org
			KDR22804.1	termitegenome.org
			KDR23248.1	termitegenome.org
Phthiraptera	*Pediculus humanus*	Phum	PHUM047320	VectorBase
			PHUM047430	VectorBase
			PHUM336650	VectorBase
			PHUM381320	VectorBase
			PHUM381330	VectorBase
			PHUM381440	VectorBase
			PHUM381550	VectorBase
			PHUM432430	VectorBase
			PHUM486750	VectorBase
			PHUM494510	VectorBase
			PHUM576820	VectorBase
			PHUM576950	VectorBase
			PHUM601890	VectorBase
Hemiptera	*Acyrthosiphon pisum*	Apis	ACYPI005277	AphidBase
			ACYPI009864	AphidBase
			ACYPI007931	AphidBase
			ACYPI003344	AphidBase
			ACYPI001792	AphidBase
			ACYPI002989	AphidBase
			ACYPI005024	AphidBase
			ACYPI006915	AphidBase
			ACYPI005018	AphidBase
			XP_003240836.1	NCBI
			XP_003245187.1	NCBI
			XP_003247564.1	NCBI
			ACYPI010066	AphidBase
			XP_008182500.1	NCBI
			XP_008184040.1	NCBI
Coleoptera	*Tribolium castaneum*	Tcas	TC002898	BeetleBase
			TC011937	BeetleBase
			TC016278	BeetleBase
			TC016280	BeetleBase
			TC008803	BeetleBase
			TC010987	BeetleBase
			TC011121	BeetleBase
			TC010977	BeetleBase
			TC013861	BeetleBase
			TC011938	BeetleBase
Hymenoptera	*Apis mellifera*	Amel	GB12176	BEEBASE
			GB19268	BEEBASE
			GB45596	BEEBASE
			GB46038	BEEBASE
			GB51247	BEEBASE
			GB51250	BEEBASE
			GB54302	BEEBASE

			GB54397	BEEBASE
			GB54399	BEEBASE
			GB54401	BEEBASE
			GB55040	BEEBASE
Diptera	*Drosophila melanogaster*	Dmel	CG11801 (Elo68beta)	FlyBase
			CG16904	FlyBase
			CG16905	FlyBase
			CG17821	FlyBase
			CG18609	FlyBase
			CG2781	FlyBase
			CG30008	FlyBase
			CG31141	FlyBase
			CG31522	FlyBase
			CG31523	FlyBase
			CG32072 (Elo68alpha)	FlyBase
			CG33110	FlyBase
			CG3971 (baldspot)	FlyBase
			CG5278	FlyBase
			CG5326	FlyBase
			CG6921 (james bond)	FlyBase
			CG8534	FlyBase
			CG9458	FlyBase
			CG9459	FlyBase
Lepidoptera	*Bombyx mori*	Bmor	XP_004924759.1	NCBI
			XP_004924772.1	NCBI
			XP_004924776.1	NCBI
			XP_004924792.1	NCBI
			XP_004931946.1	NCBI
			XP_004931947.1	NCBI
			XP_004931951.1	NCBI
			XP_012544187.1	NCBI
			XP_012544586.1	NCBI
			XP_012544587.1	NCBI
			XP_012544599.1	NCBI
			XP_012547369.1	NCBI
